# Deformable Fricke-XO-Gelatin Radiochromic Dosimeter of Ionizing Radiation and Its Applications in Quality Assurance Tests for Radiation Therapy

**DOI:** 10.3390/ma18133135

**Published:** 2025-07-02

**Authors:** Michał Piotrowski, Piotr Maras, Zbigniew Stempień, Radosław Wach, Marek Kozicki

**Affiliations:** 1 Department of Mechanical Engineering, Informatics and Chemistry of Polymer Materials, Faculty of Materials Technologies and Textile Design, Lodz University of Technology, 90-543 Lodz, Poland; michal.piotrowski@dokt.p.lodz.pl; 2 Department of Radiotherapy Planning, Copernicus Hospital, 93-513 Lodz, Poland; p.maras@kopernik.lodz.pl; 3 Institute of Textile Architecture, Faculty of Materials Technologies and Textile Design, Lodz University of Technology, 90-543 Lodz, Poland; zbigniew.stempien@p.lodz.pl; 4 Institute of Applied Radiation Chemistry, Faculty of Chemistry, Lodz University of Technology, 93-590 Lodz, Poland; radoslaw.wach@p.lodz.pl; 5 GeVero Co., 90-980 Lodz, Poland

**Keywords:** Fricke gel dosimeter, radiotherapy, 2D bolus dosimeter, flexible dosimeter, *in vivo* dosimetry

## Abstract

This work presents a Fricke radiochromic gel dosimeter with xylenol orange (XO) and a gelatin matrix modified with sorbitol. The dosimeter, combined with 2D scanning using a flatbed scanner and data processing using dedicated software packages, creates a radiotherapy dosimetry measurement system. The dosimeter reacts to ionizing radiation by changing color as a result of the formation of complexes of Fe^3+^ and XO molecules. It was characterized in terms of thermal and chemical stability and mechanical properties. The presence of sorbitol improved the mechanical and thermal properties of the dosimeter. The dosimeter maintains chemical stability, enabling its use in dosimetric applications, for at least six weeks. The dose–response characteristics of the dosimeter are discussed and indicate a dynamic dose–response of the dosimeter (up to saturation) of about 20 Gy and a linear dose–response of about 12.5 Gy. The following applications of the dosimeter are discussed: (i) as a 2D dosimeter in a plastic container for performing a coincidence test of radiation and mechanical isocenters of a medical accelerator, and (ii) for *in vivo* dosimetry as a 2D dosimeter alone and simultaneously as a bolus and a 2D dosimeter. Research has shown that the dosimeter has promise in many applications.

## 1. Introduction

Radiotherapy is a complex technique that uses state-of-the-art irradiation instruments to eradicate cancer using the properties of ionizing radiation. The success of radiotherapy involves many aspects to ensure proper quality assurance (QA) of patient treatment. In this regard, ionizing radiation dosimetry systems play a key role. Among others, a number of new 3D radiochromic and polymer gel dosimeters have been proposed [1,2,3,4]. Subsequently, they were used not only to measure the 3D radiation dose distribution and compare it with the dose distribution calculated by the treatment planning systems [1,2,3,4], but also to perform QA tests of the medical accelerators (e.g., coincidence of the radiation isocenter with the mechanical and imaging isocenter [5,6,7,8]). Based on these types of 3D dosimeters, dosimetry systems for 2D measurements have also been presented [9,10,11,12]. However, in addition to the above-mentioned tests, *in vivo* dosimetry for measuring the entrance radiation dose is also an important element of QA in radiotherapy. Currently, active detectors (diodes, MOSFETs, OSLDs) and passive detectors (radiochromic films, TLDs) are used for *in vivo* dosimetry [13]. Both types of detectors enable point and 2D dose measurements with high spatial resolution. This work is related to 2D radiochromic dosimetry systems.

The Fricke-XO-Gelatin with sorbitol was chosen as a dosimetry system in which sorbitol makes the dosimeter substance flexible. This property can be exploited to develop adaptable and deformable 2D dosimeters. The natural movement and deformation of internal organs can have a significant impact on the effectiveness of radiotherapy; hence new deformable gel dosimeters have emerged in recent years. The first of them were polymer dosimeters with a gelatin matrix inserted into a latex membrane [14] or enclosed in low-density polyethylene (LDPE) wraps [15]. The Presage-Def dosimeter was a modification of the commercial PRESAGE^®^ dosimeter (Heuris INC, Skillman, NJ, USA), in which the originally used polyolefin was replaced with a polyether, which allowed the obtaining of a flexible polyurethane matrix [16]. The latest deformable radiochromic dosimeters (LMG-silicone and FlexyDos3D) were obtained using a SYLGARD^®^ 184 Silicone Elastomer Kit (Dow Corning, Midland, MI, USA) as matrix and a leucomalachite green (LMG) dye [17,18,19].

Fricke gel dosimeters are one of the most developed groups of dosimeters. The first 3D dosimeter containing Fricke solution (acidified solution of Fe^2+^ ions) [20] introduced into a gelatin matrix was proposed in the 1980s [21]. As a result of irradiation, radiolysis of water in the dosimeter occurs. The products of radiolysis enter into the following reactions (Equations (1)–(6)) leading to the oxidation of ferrous to ferric ions.

(1) $$H^{•}+O_{2}\to {HO}_{2}^{•}$$

(2) $$H^{•}+H_{2}O\to {OH}^{•}+H_{2}$$

(3) $${HO}_{2}^{•}+{Fe}^{2+}\to {HO}_{2}^{-}+{Fe}^{3+}$$

(4) $${HO}_{2}^{-}+H^{+}\to H_{2}O_{2}$$

(5) $${OH}^{•}+{Fe}^{2+}\to {OH}^{-}+{Fe}^{3+}$$

(6) $$H_{2}O_{2}+{Fe}^{2+}\to {OH}^{-}+{Fe}^{3+}+{OH}^{•}$$

Readout of the recorded dose distribution was only possible using expensive and hard-to-access magnetic resonance imaging (MRI). A significant disadvantage of using Fricke solution in 3D dosimetry is the high diffusion coefficient of Fe^3+^ ions, which means that the dose distribution should be read as soon as possible after irradiation. The use of xylenol orange (XO) dye [22], capable of complexing Fe^3+^ ions, allowed for a reduction in the diffusion coefficient. Although the diffusion of ferric ions was still a problem [23], the introduction of the dye into the Fricke dosimeter allowed the dose distribution to be read using optical methods more readily available than MRI, such as optical computed tomography (OCT) [24]. In recent years, the use of various gel matrices such as gelatin [25,26,27], agarose [28], poly(vinyl alcohol) (PVA) [29,30,31], and Pluronic F-127 [32,33,34] has been proposed. In addition, additives such as glutaraldehyde or gellan gum added to PVA [30,35] and sucrose or glycerol added to gelatin [36] have been introduced to the matrices. The addition of 40% sucrose significantly reduced the ferric ions diffusion coefficient, but due to the optical activity of the compound, artifacts occurred during the dose distribution readout. Other disadvantages of the addition of sucrose were the lower chemical stability of the dosimeter and a long time, even up to several hours, during which the color of the irradiated gel changed. Replacing sucrose with 23% sorbitol eliminated the problem of the long time of increasing the radiochromic response of the gel [37]. Moreover, no artifacts were observed in 2D measurements. Sorbitol is an additive capable of plasticizing gelatin [38,39,40], which may enable the formation of a flexible Fricke dosimeter that can be used as a hydrogel bolus sensitive to ionizing radiation (hydrogels are promising materials for producing boluses due to their high tissue similarity and good adhesion to the skin [41]; however, they exhibit significantly worse mechanical properties than plastic and silicone boluses [41,42,43]) or a thin dosimeter for determining the dose distribution on the skin, which can be an alternative to currently used thermoluminescent dosimeters (TLDs) [44], Gafchromic^TM^ EBT films [45,46], and MOSFET detectors [47].

**Figure 1:**
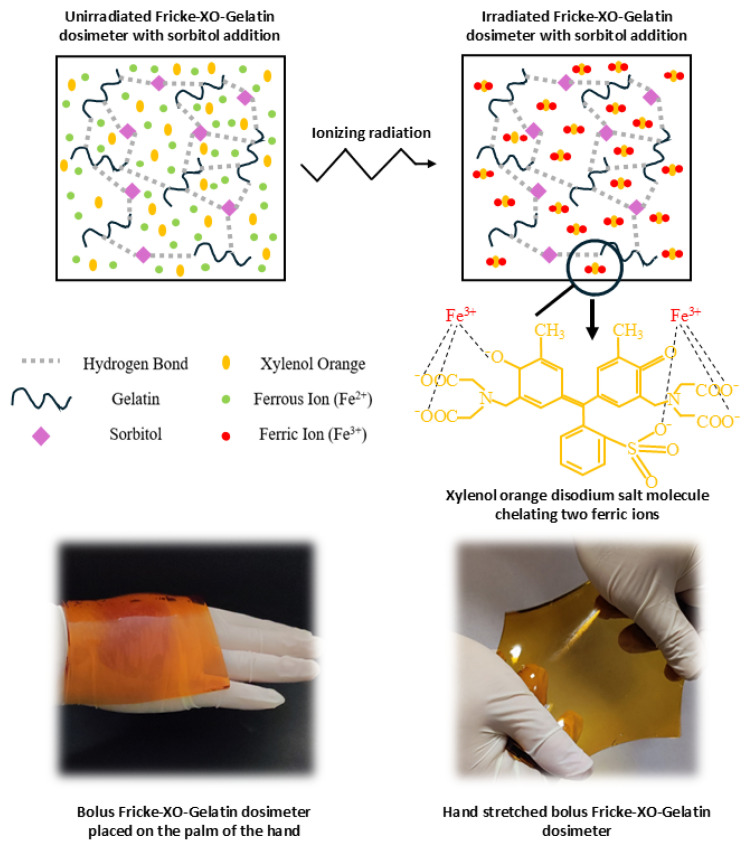
Illustration of the interactions between components and reactions occurring after irradiation of the Fricke-XO-Gelatin dosimeter with sorbitol. In addition, the final product in the form of a gel dosimeter acting as a bolus is presented.

The aim of this study was to obtain a deformable tissue-equivalent 2D radiochromic gel dosimeter characterized by high spatial resolution, acting simultaneously as a bolus and an *in vivo* dosimeter, while additionally enabling the performance of a medical accelerator test. For this purpose, an appropriate modification of the Fricke-XO-Gelatin dosimeter was made. A significant advantage of this dosimeter is that it can be analyzed with a flatbed (optical) scanner in a relatively short time after irradiation (less than 1 h), which, together with the appropriate software for fast data processing, creates a measurement system. The idea of such a system enables reactions after irradiation and interactions between components, resulting in the formation of the final product as shown in Figure 1. To achieve the objectives of this work, the thermal and mechanical properties, chemical stability, and sensitivity of the dosimeter to ionizing radiation were first investigated. Then, three examples of the dosimeter’s application in clinical practice were discussed: (i) a tool for performing a coincidence test of the accelerator’s radiation and mechanical isocenter, (ii) a hydrogel bolus capable of measuring the dose distribution, and (iii) a thin dosimeter for determining the dose distribution on the skin. The measured dose distribution with Fricke-XO-Gelatin with sorbitol was compared with calculations using a treatment planning system (TPS) and Monte Carlo (MC) simulations.

## 2. Materials and Methods

### 2.1. Fricke Gel Preparation

To prepare the Fricke-XO-Gelatin dosimeter with sorbitol, gelatin (type A, 300 Bloom, Sigma-Aldrich, Saint Louis, MO, USA) was dissolved in water at 55 °C. After complete dissolution, it was cooled down to 33 °C. Afterwards, Fricke solution, consisting of sulfuric acid (H_2_SO_4_, Chempur, Piekary Śląskie, Poland), ferrous ammonium sulfate ((NH_4_)2Fe(SO_4_)2·6H_2_O, FAS, Chempur, Piekary Śląskie, Poland), and xylenol orange disodium salt (Sigma-Aldrich, Saint Louis, MO, USA), was added. All ingredients of the Fricke solution were dissolved in 5% of final water mass. The sorbitol (Biomus, Lublin, Poland) solution prepared in 35% of final water mass was added by syringe. The syringe outlet was placed near dipole present in a beaker with gelatin to improve the mixing of all compounds. The final concentrations of all ingredients were as follows: 5.99% gelatin, 23% sorbitol, 50 mM sulfuric acid, 0.5 mM FAS, and 0.165 mM XO. Additionally, in the studies of thermal and mechanical properties, gels with the following compositions were used: 8% gelatin, 6% gelatin with 23% sorbitol, and 7.96% gelatin with Fricke solution (the concentrations of the components in the Fricke dosimeter were 50 mM sulfuric acid, 0.5 mM FAS, and 0.165 mM XO). Double-distilled water was used to prepare all samples. All reagents were weighed on a laboratory scale with an accuracy of ±0.1 mg.

### 2.2. Preparation of the Samples

**Figure 2:**
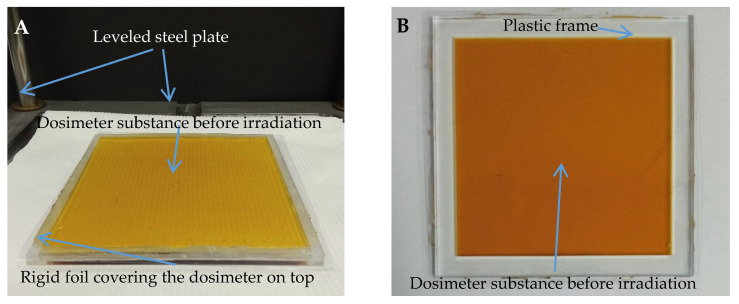
Fricke-XO-Gelatin with sorbitol dosimeter (**A**) in a cuboidal container placed on a leveled steel plate; (**B**) in a frame covered on both sides with sheets of rigid foil.

Samples for the coincidence test of mechanical and radiation isocenters were prepared by pouring the dosimeter solution into 12 cm × 12 cm × 0.3 cm poly(methyl methacrylate) (PMMA) cuboidal containers placed on a leveled stainless steel plate (Figure 2A). The filled containers were covered with a sheet of rigid foil to remove excess solution. Two steel plates were then placed on the sample. After 15 min, the plates were removed and the container with the gel was placed in a refrigerator (approximately 4 °C) for 72 h before irradiation.

A two-dimensional bolus dosimeter was prepared by pouring the dosimetric solution into a square PMMA frame with internal dimensions of 10 cm × 10 cm × 0.5 cm, which was placed on a sheet of rigid foil previously put on a leveled stainless steel plate (Figure 2B). After pouring the solution, the frame was covered with another sheet of rigid foil, and then two stainless steel plates were put on top of the sample to remove excess solution. After 15 min, the plates were removed, and the frame covered with foil was placed in the refrigerator for 72 h before irradiation. Such sample was used for experiment of verification of a treatment planning system calculated irradiation plan.

**Figure 3:**
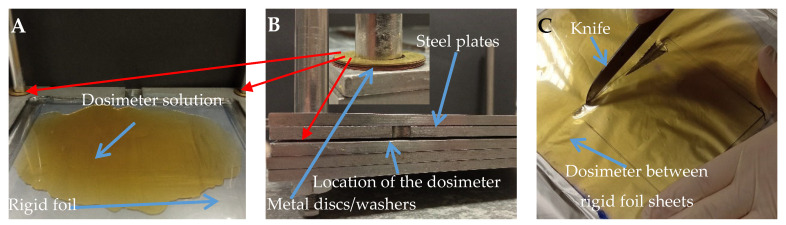
(**A**): Fricke-XO-Gelatin with sorbitol dosimeter solution poured onto a sheet of rigid foil lying on a leveled steel plate. (**B**): Dosimeter solution between two sheets of rigid foil placed on a leveled steel plate and pressed with two other plates to remove excess solution and obtain a gel layer thickness of 1 mm. The distance between the plates was controlled by metal disks placed in the corners of the plate (their location is indicated by the red arrow). (**C**): Cutting a 10 × 10 cm^2^ thin dosimeter sample using a knife.

A thin dosimeter with a gel layer thickness of 1 mm was prepared by pouring the dosimeter solution onto a sheet of rigid foil placed on a leveled steel plate (Figure 3A). The poured solution was covered with another sheet of foil and pressed with steel plates to remove excess solution and obtain a gel of the desired thickness. The distance between the plates was controlled by placing bronze disks between them (Figure 3B). After 15 min, the plates pressing the sample were removed and then the gel remaining between the two sheets of rigid foil was wrapped in aluminum foil and placed in a refrigerator for 24 h. After this time, a dosimeter sample of dimensions 10 cm × 10 cm × 0.1 cm was cut out with a sharp knife (Figure 3C). The sides of the sample were wrapped with Parafilm^®^ to protect the gel from drying out. Afterwards, the sample was kept in a refrigerator until irradiation (the total time from preparation to irradiation was 72 h). Such a sample was used for experiment of verification of a treatment planning system calculated irradiation plan.

Samples for compression strength measurements were made of 8% gelatin, 6% gelatin with 23% sorbitol, 7.96% gelatin with Fricke solution, and 5.99% gelatin with Fricke solution and 23% sorbitol (the amount of gelatin in the samples was the same; the difference in gelatin concentration between the gels is due to the addition of sorbitol and the ingredients of the Fricke solution). The solutions of the tested gels were poured into an aluminum cylindrical mold with a height and internal diameter of 2.5 cm, covered from the bottom with Parafilm^®^. After filling, the top of the mold was covered with Parafilm^®^, and the entire cylinder was placed in a refrigerator for 24 h (at approximately 4 °C). For an hour before mechanical tests, the gels were thermostated at the temperature of the room in which the measurements were performed (approximately 23 °C). The samples were removed from the molds immediately before measurement.

### 2.3. Chemical Stability Tests

The chemical stability of the Fricke-XO-Gelatin with sorbitol dosimeter was examined by pouring the dosimeter solution into poly(methyl methacrylate) (PMMA) cuvettes and storing them under different conditions, such as room temperature (approximately 25 °C) with access to light, room temperature without access to light, and low temperature (approximately 4 °C) without access to light. The color changes of the samples were measured using a UV-Vis spectrophotometer (V-530, Jasco Inc., Tokyo, Japan).

### 2.4. Thermal Analysis

An analysis of the effect of sorbitol addition on the thermal stability of the dosimeter was performed using differential scanning calorimetry (DSC, Q200, TA Instruments, New Castle, DE, USA). The instrument was calibrated for both temperature and enthalpy using indium (melting point and heat of fusion are 156.6 °C and 28.57 J g^−1^, respectively). The measurements were performed on samples of 8% gelatin, 6% gelatin with 23% sorbitol, 7.96% gelatin with Fricke solution, and 5.99% gelatin with Fricke solution and 23% sorbitol. As the stability of the Fricke solution and sorbitol sample was also evaluated over time, the sample in the Petri dish was tightly covered with several layers of Parafilm^®^ to prevent it from drying out. The set procedure comprised first heating starting typically from a temperature of 5 °C to 45 °C, then cooling down to 5 °C, followed by a second heating to 45 °C. The temperature rate in heating was 2 °C/min, as the results did not differ when the heating was performed during initial experiments with a rate of 1 °C/min.

### 2.5. Mechanical Analysis

The effect of sorbitol addition on the mechanical properties of gelatin and gelatin-Fricke gels was evaluated using a Hounsfield H10KS testing machine (Tinius Olsen, Redhill, Great Britain) through compressive strength and fatigue compressive strength tests on cylindrical gel samples (Section 2.2). For compressive strength measurements, samples were placed at the center of an aluminum table and compressed with a 40 mm diameter aluminum pin at a constant rate of 10 mm/min until failure (Supplementary Figure S1A), with force recorded using a 100 N transducer. Load–displacement data were converted into stress–strain characteristics using the equations σ = F/S (where σ is stress [N/m^2^], F is the applied load [N], and S is the initial cross-sectional area of the sample [m^2^]) and ε = l/l_0_ (where ε is strain [-], l is sample deformation [mm], and l_0_ is the initial sample length [mm]). From these curves, deformation at break, compressive force at failure, and Young’s modulus (E)—determined as the slope of the linear stress–strain region—were extracted. Fatigue compression tests were conducted by cyclically deforming the samples 100 times at a frequency of 20 cycles per minute, using a 200 N load cell (QLMH-25, Bengbu Qili Sensing System Engineering Co. Ltd., Bengbu, China) (Supplementary Figure S1B). The applied compression strain levels were 20, 40, and 60%, with corresponding compression rates of 200, 400, and 600 mm/min, respectively.

### 2.6. Irradiation

All samples were irradiated using a TrueBeam medical accelerator (TrueBeam, Varian, Palo Alto, CA, USA). To optimize the irradiation parameters for the coincidence test of radiation and mechanical isocenters, the Fricke-XO-Gelatin with sorbitol dosimeter in a cuboid container (Section 2.2) was irradiated with the following monitor units (MU): 250, 500, 750, 1000, 1500, 2000, 2500, and 4000. During irradiation, the container was placed on five slabs of the SP34 RW3 phantom (IBA, Schwarzenbruck, Germany) and covered with another two slabs with thicknesses of 1 cm and 0.5 cm. Parameters of irradiation were as follows: X-rays, 10 MV FFF (flattening filter free) beam, monitor unit rate of 2400 MU/min, high-definition multileaf collimator (HD MLC) gap of 5 mm, jaw size X: 2 cm, Y: 3 cm, gantry and collimator angle set to 0°. The sample was positioned in the isocenter of the accelerator using the LAP laser system (LAP GmbH Laser Applikationen, Lüneburg, Germany). Source to surface distance (SSD) was 100 cm.

To perform the coincidence test of radiation and mechanical isocenters, the Fricke-XO-Gelatin with sorbitol dosimeter sample was irradiated with a 2D star shot pattern with 1500 MU per beam. The irradiated sample was placed between the slabs of the SP34 RW3 phantom, in the same way as mentioned above. The irradiation parameters were as follows: X-rays, 10 MV FFF, monitor unit rate of 2400 MU/min, 5 mm gap of the HD MLC, SSD 100 cm, jaw size X: 2 cm and Y: 20 cm, collimator angle set at 0°, 90°, 150°, 240°, and gantry set to 0°. The container was placed in the isocenter of the accelerator using the LAP laser system (LAP GmbH Laser Applikationen, Lüneburg, Germany).

Bolus and thin dosimeter calibration was performed by irradiating two samples of each type. The following doses were applied: 2.5, 5, 7.5, 10, 12.5, 15, 17.5, and 20 Gy. The dosimeters were placed on fifteen slabs of the SP34 RW3 phantom, each 1 cm thick, and then six slabs of the phantom with a total thickness of 4.7 cm and 4.9 cm, respectively, were placed on them. During irradiation, the gels remained in a PMMA frame covered with a rigid foil. The following irradiation settings were applied: X-rays, 10 MV FFF, monitor unit rate of 2400 MU/min, jaw size of X: 3 cm, Y: 3 cm, gantry and collimator set to 0°. The accelerator isocenter was set on the center of the dosimeter, SSD 95 cm, and the surface of the dosimeter was perpendicular to the central axis of the radiation beam. The required number of MUs was achieved after the dosimetric measurements were performed with the aid of an ionizing chamber (CC04) with an electrometer (Dose-1), both IBA.

Verification of the treatment plans generated in the Eclipse Treatment Planning System (TPS) (v. 16.1, Varian Medical Systems, Palo Alto, CA, USA, Acuros External Beam v. 16.1.0, dose grid: 1 × 1 × 1 mm^3^) was performed using bolus and thin Fricke-XO-Gelatin with sorbitol dosimeters. Additionally, one bolus dosimeter was irradiated with doses of 2.5, 5, 7.5, and 10 Gy to check the reproducibility of the gel dosimeter. The irradiation conditions were identical as that for calibration. The second bolus sample remaining in the PMMA frame was placed on the surface of the SP34 RW3 phantom with a thickness of 20 cm and irradiated with 750 MU per beam according to the treatment plan (no irradiation was performed immediately on patients in this study). The irradiation conditions were as follows: X-rays, 10 MV FFF, monitor unit rate 2400 MU/min, field size X: 5 cm Y: 5 cm, gantry and collimator angle 0°. The SSD set on the phantom surface was equal to 100 cm. The thin dosimeter sample was placed on the surface of the 20 cm thick SP34 RW3 phantom and covered with a 15 cm × 15 cm × 1 cm Bolx with skin bolus (CQ Medical^TM^, Avondale, PA, USA). On the sample, one 5 cm × 5 cm field was irradiated with 2000 MU per beam, and three 2 cm × 2 cm fields were irradiated with 400, 800, and 1200 MU per beam. The remaining irradiation conditions were the same as those for the bolus dosimeter. Measured dose distributions with Fricke-XO-Gelatin with sorbitol were compared with dose distributions calculated using TPS and myQA iON (v. 2.1.0 IBA Dosimetry Gmbh, Schwarzenbruck, Germany, SciMoCa, dose grid: 1 × 1 × 1 mm; MC simulations). Scans of the experimental system (phantom with gel dosimeter) used to perform the calculations were obtained using Somatom Sensation Open CT scanner (Siemens AG Medical Solutions, Erding, Germany).

### 2.7. Readout and Data Processing

Irradiated dosimeters were scanned using an HP Scanjet G3010 flatbed scanner (Hewlett-Packard, Palo Alto, CA, USA) operating at the following settings: resolution of 150 dpi, brightness = 0, contrast = 0. To observe changes occurring in the irradiated samples, dosimeter scanning was repeated within 24 h of irradiation. Samples were stored at approximately 20 °C. During scanning, the container with the dosimeter was covered with three sheets of white paper with a grammage of 120 g/m^2^ (POL Effect, International Paper, Kwidzyn, Poland). The polyGeVero-CT software package [48,49] (v. 1.2, GeVero Co., Lodz, Poland) was used to perform the calculations related to the coincidence test of radiation and mechanical isocenters. The comparison of the measured dose distribution with the calculations performed using TPS and the results of the Monte Carlo simulation was performed with the aid of the polyGeVero software package [50] (v. 2.0, GeVero Co., Lodz, Poland). The comparison was performed by calculating the local gamma index distribution (3% dose difference (DD) and 3 mm distance-to-agreement (DTA) criteria), comparing the dose profiles and the isodoses.

The steps performed after sample preparation by irradiation, scanning, and data processing to calculate the calibration and compare the measured dose distribution with the Fricke-XO-Gelatin with sorbitol dosimeter with the calculated or simulated dose distributions using TPS or myQA iON/MC simulations are presented in Supplementary Figure S2.

## 3. Results and Discussion

### 3.1. Thermal Properties of the Fricke-XO-Gelatin Dosimeter with Sorbitol

Thermograms for the Fricke-XO-Gelatin dosimeter with sorbitol that were obtained using DSC are shown in Supplementary Figure S3A. Gelatin solutions form solid gels upon cooling due to formation of hydrogen bonds between its chains [40]. The gel of 8% gelatin melts at c.a. 34.8 °C (the peak transition temperature). After the addition of Fricke’s solution to gelatin, its phase transition temperature is lower by about 3 °C upon heating. Despite the minor change in the gelatin concentration (7.96% after the addition of the Fricke solution components), this decrease in the phase transition temperature is due to the low pH (about 1.4) of the Fricke’s solution containing sulfuric acid. In a strongly acidic environment, gelatin undergoes partial hydrolysis, which leads to a weakening of the gel strength and, consequently, lowers the sol–gel transition temperature [38,40]. The addition of sorbitol leads to an increase in the phase transition temperature of both plain gelatin and gelatin with Fricke solution by approximately 0.7 °C and 1.5 °C, respectively. High concentration of sorbitol in gelatin increases its melting point by stabilizing its structure [38]. In the system with Fricke solution, the presence of sorbitol has a protective effect on gelatin structure by formation of additional sorbitol–gelatin and sorbitol–sorbitol bonds. Those may undergo hydrolysis prior to the gelatin–gelatin hydrogen bonds rupture [40]. However, when pH is low enough, the protective effect of sorbitol may be insufficient, which can result in the destruction of hydrogen bonds between gelatin polypeptide chains and, eventually, even the hydrolyzation of these macromolecules. The phase transition temperature of the dosimeter with sorbitol decreased by around 1.2 °C in 12 days (Supplementary Figure S3B). After this time, the sol–gel transition temperature stabilizes at approximately 32.1 °C, which is about 0.3 °C higher than melting temperature of the dosimeter without sorbitol after just four hours following preparation (Supplementary Figure S3A). This indicates that the addition of sorbitol slightly improves the thermal properties of the Fricke-XO-Gelatin dosimeter.

### 3.2. Mechanical Properties of the Fricke-XO-Gelatin Dosimeter with Sorbitol

The representative stress–strain characteristics of the tested materials are presented in Supplementary Figure S4A. The dependence of relative stress (σ_rel_ [%])—calculated as the percentage ratio of the stress at maximum deformation after n compression cycles (n being a natural number from 1 to 100) to the stress at maximum deformation after the first cycle—is shown in Supplementary Figure S4B–D for different strains (20–60%). Additionally, the dependence of stress as a function of time is presented in Supplementary Figure S4E–G. The mean values of Young’s modulus, deformation at break, and compressive strength of the tested gels are summarized in Supplementary Table S1. As shown in Supplementary Figure S4A, the stress–strain characteristics of the gelatin-based gels exhibit a nonlinear mechanical response, with stress increasing gradually at low strain and sharply rising beyond a strain of 0.4. Both sorbitol and Fricke’s solution significantly influence the mechanical properties of gelatin, albeit in different ways. Sorbitol enhances the gel’s compressive strength while simultaneously lowering Young’s modulus and increasing deformation at break, particularly under high-strain conditions. This effect is attributed to sorbitol’s plasticizing role, which disrupts hydrogen bonding between gelatin macroparticles, increases polymer chain mobility, and consequently reduces material stiffness [51,52]. In contrast, the addition of Fricke’s solution reduces the gel’s compressive strength, with the effect being most pronounced in samples lacking sorbitol. Additionally, Fricke’s solution lowers Young’s modulus and slightly increases deformation at break. At high strain levels, all tested gels exhibit strain hardening, characterized by a rapid increase in stress before failure. These findings highlight the interplay between sorbitol and Fricke’s solution in modulating the mechanical behavior of gelatin-based gels, balancing strength, flexibility, and stiffness. During fatigue tests, the addition of sorbitol led to a noticeable increase in the stress drop at maximum deformation when the samples were compressed by 20%. This suggests that sorbitol enhances the material’s plasticity, allowing it to accommodate cyclic deformation with greater structural adaptability. At 40% compression, the stress drop observed between gelatin and gelatin–sorbitol samples became comparable, indicating that at moderate deformation levels, both materials exhibited similar mechanical responses. However, under 60% compression, a significant difference was observed: pure gelatin fractured after only 18 cycles, while the gelatin–sorbitol sample remained intact throughout the entire test duration. This indicates that sorbitol improves the material’s resistance to cyclic loading, likely due to its plasticizing effect, which enhances the mobility of gelatin chains. The increased chain mobility allows the gel to reorganize under cyclic compression, delaying the onset of structural failure. In contrast, the rigid hydrogen-bonded network in pure gelatin lacks this flexibility, leading to progressive bond breakage and eventual material failure under sustained high-strain conditions. The presence of Fricke’s solution significantly reduced the stress at maximum strain across all tested compression levels in both gelatin and gelatin–sorbitol samples. This effect was particularly pronounced at 60% strain, where both gels containing Fricke’s solution exhibited rapid failure, with gelatin breaking after 22 cycles and gelatin–sorbitol after 17 cycles. The earlier failure of the gelatin–sorbitol sample in this case suggests that while sorbitol provides mechanical reinforcement under normal conditions, it is insufficient to protect the gel structure in a highly acidic environment. This behavior aligns with previous observations on the impact of Fricke’s solution on gelatin’s mechanical properties, where the low pH hydrolyzes hydrogen bonds between polypeptide chains, weakening the overall gel structure. Although sorbitol can stabilize intermolecular interactions to some extent, its protective effect is insufficient to counteract the degradation induced by acid hydrolysis. Given these findings, strategies to mitigate the adverse effects of acidity on the mechanical properties of gelatin-based gels should be considered. One potential approach is the use of alternative radiation-sensitive components, such as tetrazolium salts [53], which may provide similar dosimetric properties without compromising gel stability. Additionally, considering that bolus materials should ideally exhibit a Young’s modulus comparable to human soft tissue (ranging from 10^4^ to 10^9^ Pa) [41], the Fricke-XO-Gelatin with sorbitol formulation falls within this range but is positioned near the lower limit. This suggests that while the material retains some elasticity, further optimization may be required to enhance its mechanical robustness. Moreover, the gel’s low pH could pose challenges to direct application on human skin, potentially leading to irritation or discomfort. To address these concerns, it would be necessary to explore packaging solutions or additional structural modifications that improve both the mechanical durability and biocompatibility of the bolus material.

### 3.3. Chemical Stability of the Fricke-XO-Gelatin Dosimeter with Sorbitol

**Figure 4:**
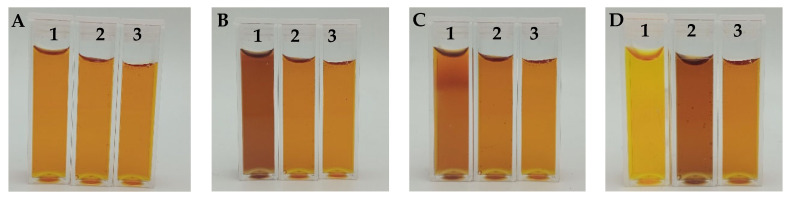

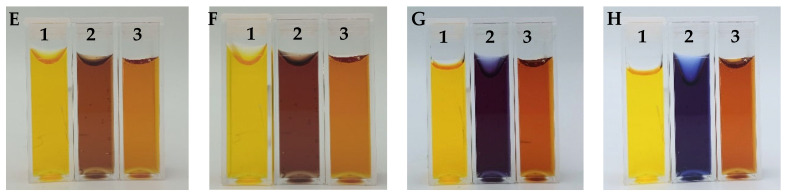
Photographs of Fricke-XO-Gelatin with sorbitol samples stored under different conditions. The numbers on the top of the cuvettes indicate storage conditions (1: table, room temperature (approximately 25 °C), light access; 2: cabinet, room temperature, no light access; 3: refrigerator, temperature approximately 4 °C, no light access). Photographs were taken at the following times from preparation: 0 h (**A**), 24 h (**B**), 48 h (**C**), 144 h (**D**), 216 h (**E**), 2 weeks (**F**), 4 weeks (**G**), and 6 weeks (**H**).

**Figure 5:**
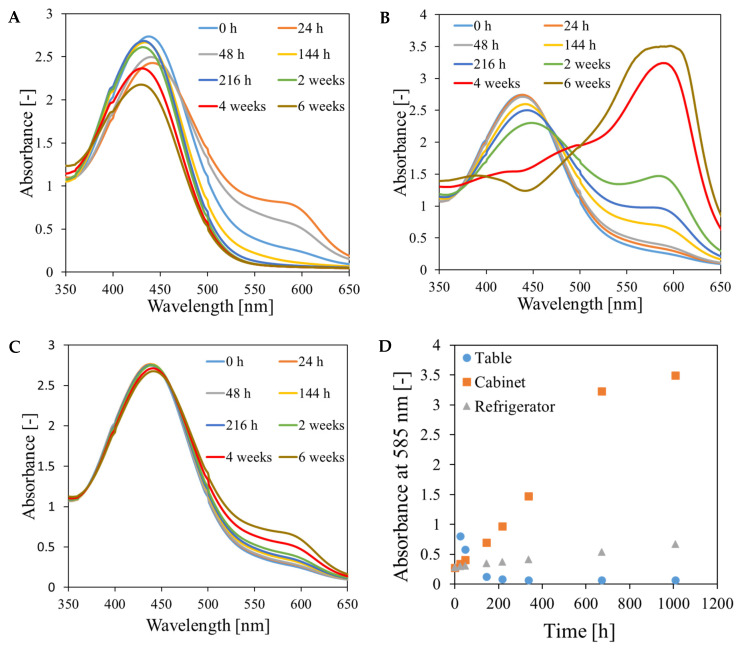
Absorption spectra of the Fricke-XO-Gelatin with sorbitol dosimeter stored under the following conditions: table, room temperature (approximately 25 °C), access to light (**A**); cabinet, room temperature, no access to light (**B**); refrigerator, temperature approximately 4 °C, no access to light (**C**). The change in the absorbance value at 585 nm during storage of the dosimeter is shown in (**D**).

The chemical stability of Fricke-XO-Gelatin with sorbitol was assessed for samples stored under various conditions (Section 2.3). During the observations, photographs were taken (Figure 4), and measurements of the samples were performed using a UV-Vis spectrophotometer (Figure 5). The dosimeter stored on the laboratory table (marked in Figure 4 as 1) darkened significantly to brown after only 24 h of storage (Figure 4B). After another 24 h, the sample lightened to orange (Figure 4C) and continued to lighten over time, changing color to yellow (Figure 4D). Such changes in the sample result from the absorption of too high doses of UV radiation [54]. The dosimeter stored in the cabinet (marked as 2) retained its initial color for 48 h after preparation (Figure 4A–C). After 144 h of storage, the gel darkened to dark orange (Figure 4D). Further darkening of the sample continued over time. After 216 h since preparation, the dosimeter turned brown (Figure 4E), and after 4 weeks, it became purple (Figure 4G), indicating complete oxidation of the ferrous ions present in the dosimeter. The refrigerated sample (marked as 3) did not visibly change color within 2 weeks after preparation (Figure 4A–F). After 1 month since preparation (Figure 4G), the gel darkened to an orange color that persisted until the end of the 6-week observation period (Figure 4H).

The absorption spectra of the samples obtained from spectrophotometric measurements (Figure 5) consist of two bands in the wavelength range of approximately 350–525 nm and 525–650 nm. The spectra change during sample storage. These, for the sample stored on the table (Figure 5A), correspond to the observations made on the basis of the photographs (Figure 4, sample 1). A distinct darkening of the sample in the spectra can be observed as an increase in the absorption maximum at 585 nm and a simultaneous decrease in the absorption maximum at 440 nm. During storage, there is a decrease in the absorption at 585 nm and a hypsochromic shift of the absorption maximum from 440 nm to 430 nm, which is visible in the photographs as a change in the color of the gel from orange to yellow. The gradual darkening and color change from orange to purple observed for the dosimeter stored in the cabinet can be seen in the spectra (Figure 5B, sample 2) as an increase in the absorption maximum at 585 nm and a simultaneous decrease in the maximum at 440 nm. In the pictures of the sample stored in the refrigerator (Figure 4, sample 3), no color changes were observed within two weeks of preparation. However, in the spectra (Figure 5C) within 14 days of storage of the gel, a slight increase in the absorption maximum at 585 nm is visible. The absorption maximum at 440 nm almost did not change during this time. Further increase in the absorption maximum after 4 and 6 weeks of preparation is less significant compared to the changes occurring in the samples stored on the table and in the cabinet (Figure 5D). The conducted experiment indicated that the Fricke-XO-Gelatin with sorbitol dosimeter should be stored in the refrigerator. Even under optimal storage conditions, changes related to the oxidation of ferrous ions occur in the gel, but these are negligible enough to successfully use the dosimeter (stored in the refrigerator for several weeks) in applications. For this purpose, before performing measurements, the dosimeter should be calibrated using a gel with the same storage history as the one used for the measurements.

### 3.4. Dosimeter Response to Ionizing Radiation

Before testing the Fricke-XO-Gelatin with sorbitol dosimeter in various applications, such as for the test of coincidence of radiation and mechanical isocenter, it was necessary to investigate its dose response to ionizing radiation, including linear response, dynamic response (response of the dosimeter up to saturation), and to derive calibration relations for both linear and dynamic dose response. The dose sensitivity parameter is also extracted from the linear calibration relation as a slope parameter. The irradiated dosimeter samples are then scanned with a flatbed scanner and the images are resolved into the red, green, and blue channels of the RGB color model to determine the channel that contributes most to the color change. The results obtained are discussed below.

**Figure 6:**
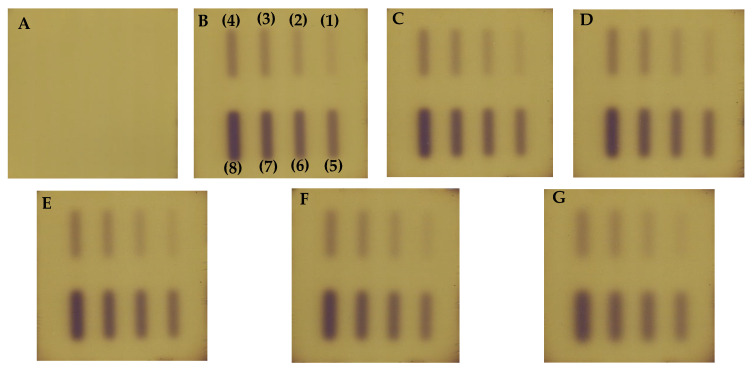
The scans of the Fricke-XO-Gelatin with sorbitol sample before (**A**) and after (**B**–**G**) irradiation with 250 (1), 500 (2), 750 (3), 1000 (4), 1500 (5), 2000 (6), 2500 (7), and 4000 (8) MU (as shown in (**B**)). Sample scanning was performed at the following times after irradiation: 113 min (**B**), 234 min (**C**), 345 min (**D**), 423 min (**E**), 568 min (**F**), and 1437 min (**G**).

A sample of the Fricke-XO-Gelatin with sorbitol dosimeter in a cuboidal container (Section 2.2) was irradiated in the range of 250 to 4000 MU. Images of the samples scanned before and until 24 h after irradiation using a flat-bed HP Scanjet G3010 scanner are presented in Figure 6. The irradiated areas in the form of stripes of dimensions 0.5 cm × 4 cm changed color from orange to purple. A purple color was also visible at the edges of the sample, probably from contact of the gel with the glue used to attach the walls of the container to the plate constituting its bottom. A similar effect was observed for the Fricke-XO-Pluronic F-127 dosimeter [9]. In the Pluronic matrix dosimeter, however, the purple region at the edges increased with time towards the center of the sample, while for gelatin with sorbitol, the area did not change with time. This effect on the sample edges had no impact on further image analysis. The irradiated stripes became more blurred and wider with time due to the diffusion of ferric ions; hence, the sample should be scanned as soon as possible after irradiation.

**Figure 7:**
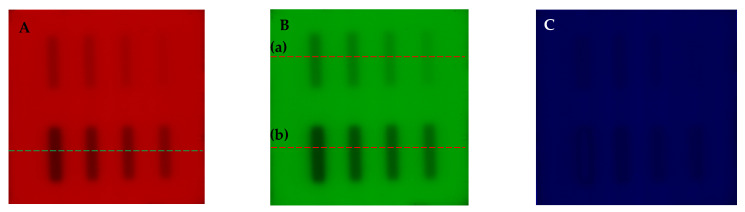

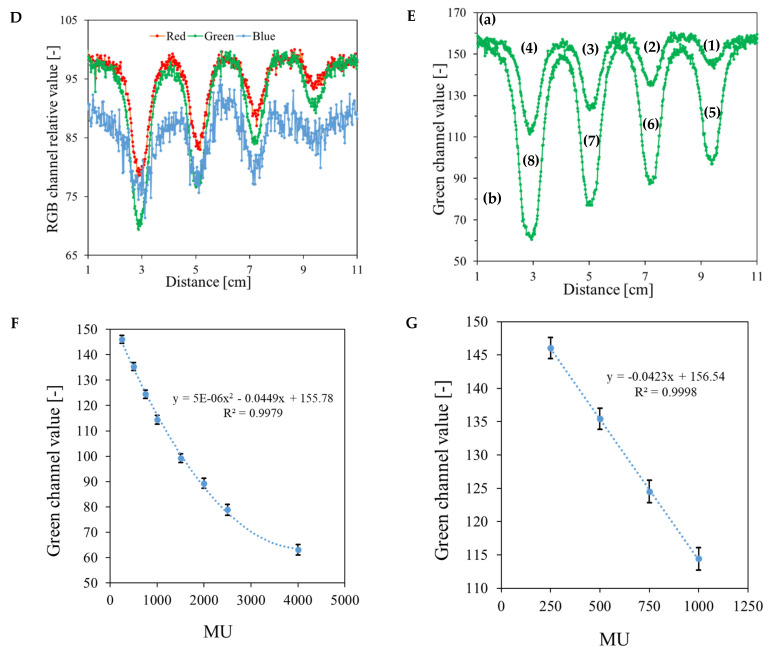
Irradiation response analysis of the Fricke-XO-Gelatin with sorbitol. (**A**–**C**): images obtained by decomposing a scan of the dosimeter sample taken 113 min (Figure 6B) after irradiation into red (**A**), green (**B**) ((a) and (b) denote positions of profiles presented in **E**), and blue (**C**) channels of the RGB color model. (**D**): Red, green, and blue color profiles determined across the bottom of the irradiated sample (indicated by the green dashed line in (**A**)). (**E**): Green channel value profiles determined across the top and bottom of the sample (indicated by the red dashed lines (a) and (b) in (**B**)). Profiles marked with a number in (**E**) correspond to the stripes marked with the same numbers in Figure 6B. (**F**): The relation of the green channel value versus monitor units. (**G**): The same relationship as in F but in the range of the linear response of the dosimeter.

The obtained scans of the Fricke-XO-Gelatin with sorbitol dosimeter samples are presented in Figure 7A–C after being resolved into red, green, and blue channel of the RGB color model. Across the images obtained by decomposition into channels, perpendicular to the longer side of the irradiated stripes, profiles were determined (Figure 7D,E). Based on the profiles, it was found that the green color changes the most with the sample irradiation, hence further analysis of the Fricke-XO-Gelatin with sorbitol dosimeter images was performed for this channel. The relationship between the green channel value (calculated as the mean of the irradiated regions) and the number of monitor units (MU) is shown in Figure 7F,G. From these figures the following were extracted: (i) the dynamic response of the dosimeter is up to 4000 MU where upon exceeding this value, it clearly starts to saturate; (ii) the linear response is up to about 1000 MU; (iii) the calibration relations have been derived and can be seen in Figure 7F,G for the dynamic and linear response, respectively; (iv) the irradiation sensitivity is −0.0423 MU^−1^, as extracted from the linear response of the dosimeter. The dosimeter was not irradiated below 250 MU; therefore, no MU threshold that causes the primarily noticeable color change could be extracted.

The profiles determined across the irradiated areas 113, 234, and 1437 min after irradiation are presented in Supplementary Figure S5. The width of the profiles and the green color value did not change between 113 min and 234 min of irradiation. This shows the advantage of sorbitol over sucrose, the addition of which to the Fricke-XO-Gelatin dosimeter increased the dosimeter response to ionizing radiation even up to several hours after irradiation [36]. Similarly, for the two-dimensional Fricke-XO-Pluronic F-127 dosimeter, the green channel value decreased between 44 min and 169 min of irradiation [9]. Moreover, in this time interval, there was a significant broadening of the determined profiles, which indicates a higher diffusion coefficient of the Fricke dosimeter in the Pluronic matrix than in gelatin with sorbitol. Between 234 min and 1437 min, the profiles determined for the tested dosimeter widened and the value of the green channel increased, which is caused by the diffusion of ferric ions. Therefore, the sample should be scanned no later than 4 h after irradiation.

In summary, Fricke-XO-Gelatin with sorbitol responds to each delivered MU and is stable for at least 4 h after irradiation. To clearly observe changes in the dosimeter after irradiation, it is recommended to irradiate the dosimeter above 250 MU and below 2000 MU. The period of stability after irradiation is sufficient for scanning the dosimeter in 2D using a flatbed scanner or in 3D using optical computed tomography, provided that the 3D scanner is located near the irradiation unit.

### 3.5. Bolus and Thin Dosimeter Calibration

**Figure 8:**
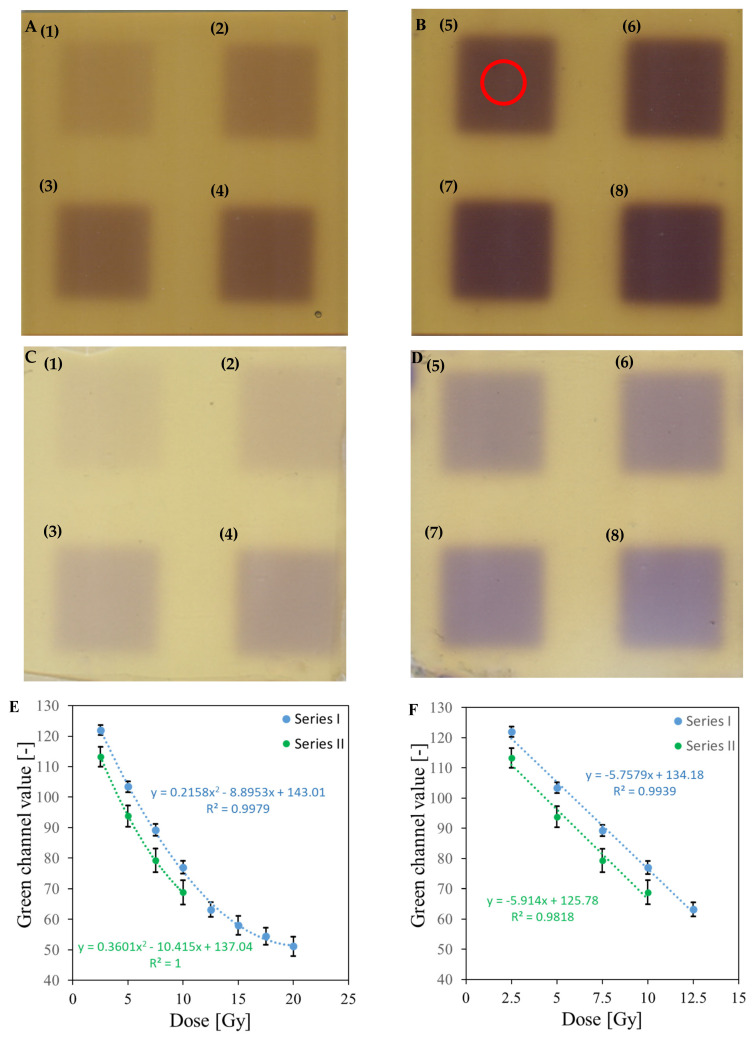

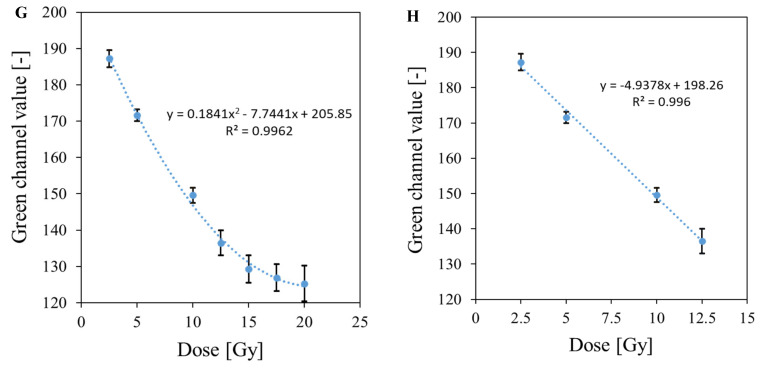
Dose response of Fricke-XO-Gelatin with sorbitol in form of bolus and thin gel samples. (**A**,**B**): Scans of the bolus samples irradiated with the following doses: 2.5 (1), 5 (2), 7.5 (3), 10 (4), 12.5 (5), 15 (6), 17.5 (7), and 20 Gy (8). The red circle in (**B**) limits the area used to determine the green channel mean value. (**C**,**D**): Scans of the thin dosimeters. Numbers 1–8 denote to the same doses as in (**A**,**B**). Green channel value versus dose relationship for bolus and thin dosimeter is presented in (**E**) and (**G**), respectively. (**F**,**H**) refers to the same relation for a narrower range of doses and using linear regression. F refers to bolus and H refers to the thin dosimeter.

The scans of the bolus and thin dosimeter samples irradiated with doses of 2.5–20 Gy are shown in Figure 8A–D. The unirradiated bolus is dark yellow, while the unirradiated thin dosimeter is light yellow. This difference in color intensity results from the different thickness of the gel layer, which is 5 mm for the bolus and 1 mm for the thin dosimeter. Both dosimeters change color to purple after irradiation, which becomes more intense with increasing radiation dose. Color inhomogeneities in the form of vertical stripes of different brightness are slightly visible in the scans. This occurs probably due to the insufficient quality of the scanner used and the impact of the structure of foils covering the gels. Figure 8E–H shows the value of the green channel as a function of dose for the scanned samples. The values presented in the graphs were determined as the mean value of the green channel in the central part of the irradiated area limited by a circle with a diameter of 1.5 cm (indicated by the red circle in Figure 8B). Calibration of the bolus dosimeter was performed in the range of 2.5–20 Gy (Series I in Figure 8E,F) and repeated in the range of 2.5–10 Gy (Series II in Figure 8E,F). These calibrations for both Series were performed for Fricke-XO-Gelatin with sorbitol, using fresh and aged stock solution to investigate the impact of storage on the dosimeter dose response. The calibration curves obtained differ from each other. The green channel values for the same doses are ~10% lower in Series II than in Series I. Furthermore, the standard deviation of the values in Series II is higher. The differences obtained are likely due to partial self-oxidation of the ferrous ions. The same stock Fricke solution was used to prepare both calibration samples; however, the solution was two weeks older at the time of the repeated calibration. Moreover, the irradiation of the second calibration sample was performed three days after preparation, whereas for the first calibration, a sample two days after preparation was used. The thin dosimeter is characterized by higher values of the standard deviation of the determined green channel values, which can be the result of the inhomogeneous thickness of the gel layer due to the use of a non-optimal preparation method as follows: Before pressing with a steel plate, the poured dosimeter solution was covered with a sheet of foil. During the covering with foil, it was not possible to control the thickness of the gel, which could lead to excessively strong pressing of the solution in some places. In addition, cutting out the sample with a knife possibly affected the gel thickness, especially at the sample boundaries. The obtained results indicate the need to improve the applied method of obtaining the thin dosimeter. In summary, the following characteristics are important for further applications of such samples: (i) Fricke-XO-Gelatin with sorbitol, both bolus and thin gel, respond dynamically to irradiation up to 20 Gy, with the dosimeters becoming saturated upon exceeding this dose; (ii) the linear dose response is up to about 12.5 Gy; (iii) the threshold dose was not estimated because the samples were not irradiated below 2.5 Gy; (iv) the calibration equations are given in Figure 8E–H, with the slope parameters of the linear regression indicating the dose sensitivities of the samples; (v) the calibration relations are dependent on the freshness of the Fricke stock solution and self-oxidation of Fe2+ ions. Consequently, caution should be exercised when designing the application study. For example, if the dosimeter is used for 3D dose verification in radiotherapy dosimetry, both the calibration dosimeter and verification gel samples should be used from the same stock solution with the same storage history. Table 1 compares the radiation properties of thin and thick Fricke-XO-Gelatin with sorbitol with other deformable dosimeters.

### 3.6. Applications of the Fricke-XO-Gelatine–Sorbitol Dosimeter

#### 3.6.1. Coincidence Test of Radiation and Mechanical Isocenters

**Figure 9:**
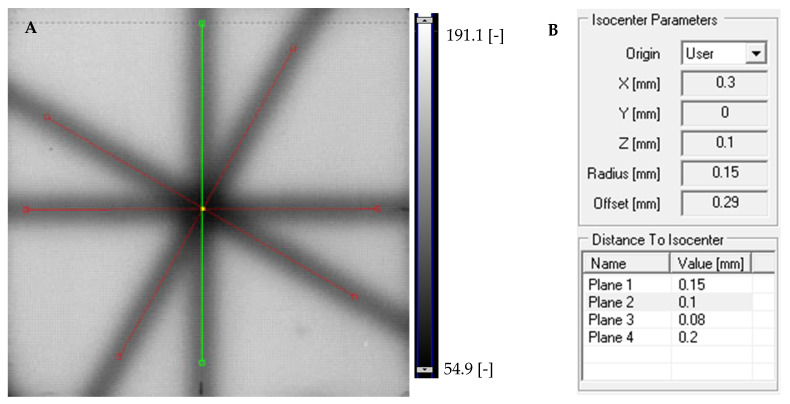
Application of Fricke-XO-Gelatine with sorbitol to the coincidence test of radiation and mechanical isocenters for the TrueBeam (Varian, Las Vegas, NV, USA) medical accelerator. (**A**): Green channel image obtained by splitting the sample scan into RGB color model channels viewed in the polyGeVero-CT software package (GeVero Co., Poland). Red and green lines are specific tools for determining the isocenter, while the yellow dot indicates the location of the radiation isocenter. (**B**): Results of the test, isocenter parameters: location, radius, and offset (distance of the radiation isocenter to the mechanical isocenter of the medical accelerator).

Fricke-XO-Gelatin with sorbitol 2D dosimeter was used to perform the coincidence test of radiation and mechanical isocenters of the TrueBeam medical accelerator, which is used in radiotherapy. This was possible due to favorable dose–response characteristics and satisfactory stability of the dosimeter both before and after irradiation, as reported above. The following steps were performed: (i) 2D star shot irradiation of the Fricke-XO-Gelatin with sorbitol in a cuboidal container with 1500 MU per beam; (ii) scanning the sample (109 min after irradiation) using the HP Scanjet G3010 scanner; (iii) calculations related to the test based on the 2D images of the irradiated dosimeter sample using the dedicated tools of the polyGeVero-CT software package. The sample image was decomposed into red, green, and blue channels of the RGB color model. The green channel image selected for analysis is presented in Figure 9A. As can be deduced from this figure, the star-shaped color changes after irradiation are extremely visible to the naked eye, and a strong signal in the irradiated part was recorded. For this reason, the calculations in polyGeVero-CT were fast, easy, and with less problems; no filtering of images or other operations on image, before the coincidence test calculations, were needed. The results of the calculations (isocenter radius and offset distance between the radiation and mechanical isocenters) are presented in Figure 9B. The calculated isocenter radius value was 0.15 mm, and the offset was 0.29 mm. Both values are within the tolerance limits for the star shot measurement: ±1 mm [55]. The total standard uncertainty of the radiation and mechanical isocenter coincidence test performed with using a tool consisting of a Fricke-XO-Gelatin dosimeter with sorbitol in a PMMA container, a flatbed scanner, and a polyGeVero-CT software package is 1.06 mm. The method for determining the total uncertainty is presented elsewhere [9,10].

#### 3.6.2. Treatment Planning Verification: Fricke-XO-Gelatine (Thin Gel)

**Figure 10:**
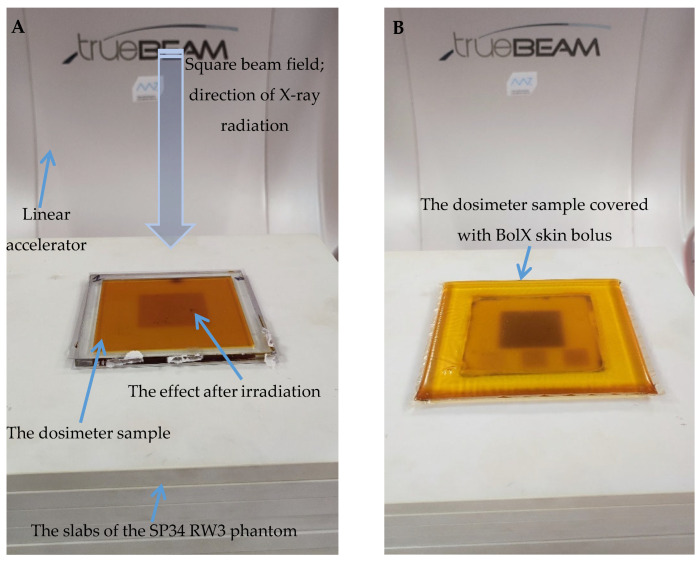
Fricke-XO-Gelatine with sorbitol samples after irradiation with a TrueBeam accelerator (Varian, USA). (**A**): Thick dosimeter located on the slabs of the SP34 RW3 phantom. (**B**): Thin dosimeter placed on SP34 RW3 phantom. The sample was covered with a 10 mm thick BolX skin bolus.

Verification of a treatment plan (generated using a TPS) was performed using a thick (bolus) (Figure 10A) and thin (Figure 10B) dosimeter. In addition, Monte Carlo simulations, using myQA iON, were performed and the results were compared to the measured dose distribution by the dosimeters and to the TPS calculated data. This section presents the results for the thin dosimeter, whereas the next section is for the thick bolus dosimeter. During irradiation, the samples rested on a 20 cm SP34 RW3 phantom. The thin dosimeter was covered with a 10 mm thick BolX with a skin bolus (Figure 10B). In this experiment, the approach to using the thin dosimeter and thick bolus was that the thin dosimeter should be used in conjunction with boluses commonly used in radiotherapy and should be covered with one to study the dose distribution on the skin surface, while the thick bolus serves simultaneously as both a bolus and a 2D dosimeter. These experiments are considered as preliminary *in vivo* dosimetry studies, in which the dose distribution of ionizing radiation is measured at the point of radiation entry into the patient’s skin.

**Figure 11:**
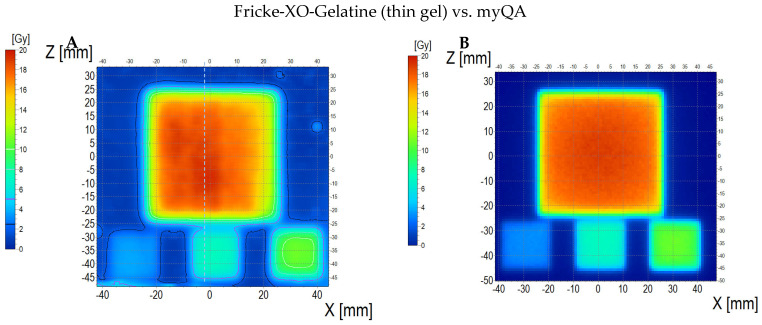

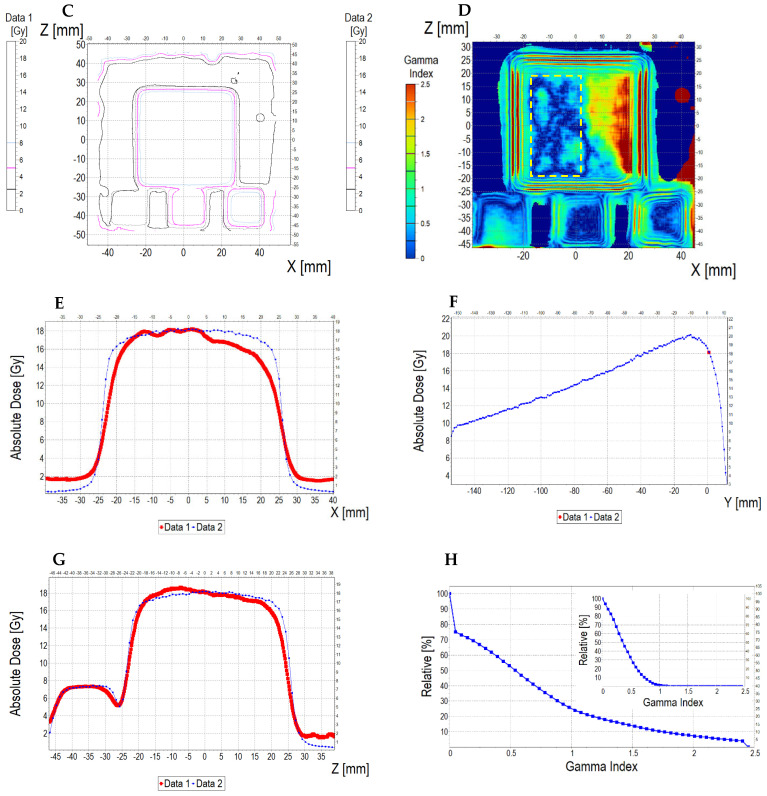
Comparison of dose distribution measured with Fricke-XO-Gelatine 2D dosimeter (thin gel) and calculated with myQA iON/MC simulations. (**A**) and (**B**): ZX planes of dose distributions for Fricke-XO-Gelatine and myQA iON/MC simulations, respectively; (**C**): superimposition of isodoses; (**D**): gamma index (DD = 3%, DTA = 3 mm); (**E**–**G**): profiles along Z, Y, and Z axes (positi on of profiles is presented in (**A**) by the lilac dashed lines); (**H**): gamma index histogram. Inset in (**H**): a gamma index histogram for a ROI indicated in (**D**) with the yellow dashed line; 1.2% pixels above gamma index = 1.

**Figure 12:**
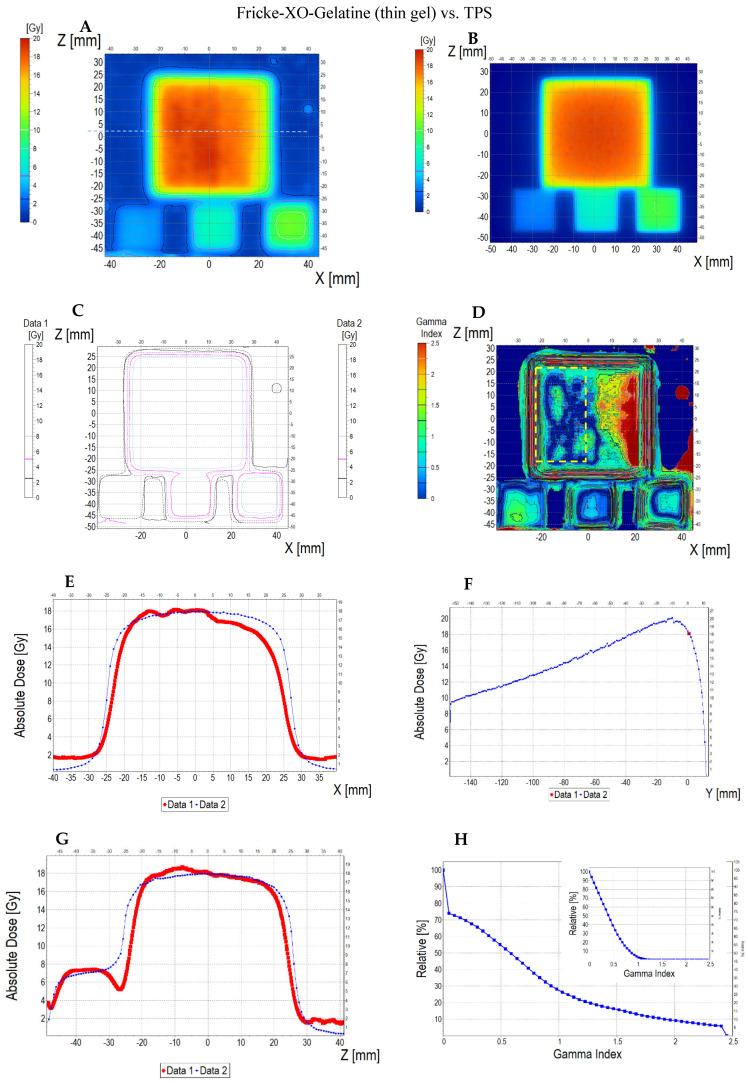
Comparison of dose distribution measured with Fricke-XO-Gelatine 2D dosimeter (thin gel) (Data 1) and calculated with TPS (Data 2). (**A**) and (**B**): ZX planes of dose distributions for Fricke-XO-Gelatine and TPS, respectively; (**C**): superimposition of isodoses; (**D**): gamma index (DD = 3%, DTA = 3 mm); (**E**–**G**): profiles along Z, Y, and Z axes (position of profiles is presented in (**A**) by the lilac dashed lines); (**H**): gamma index histogram. Inset in (**H**): a gamma index histogram for a ROI indicated in (**D**) with the yellow dashed line; 3.3% pixels above gamma index = 1.

**Figure 13:**
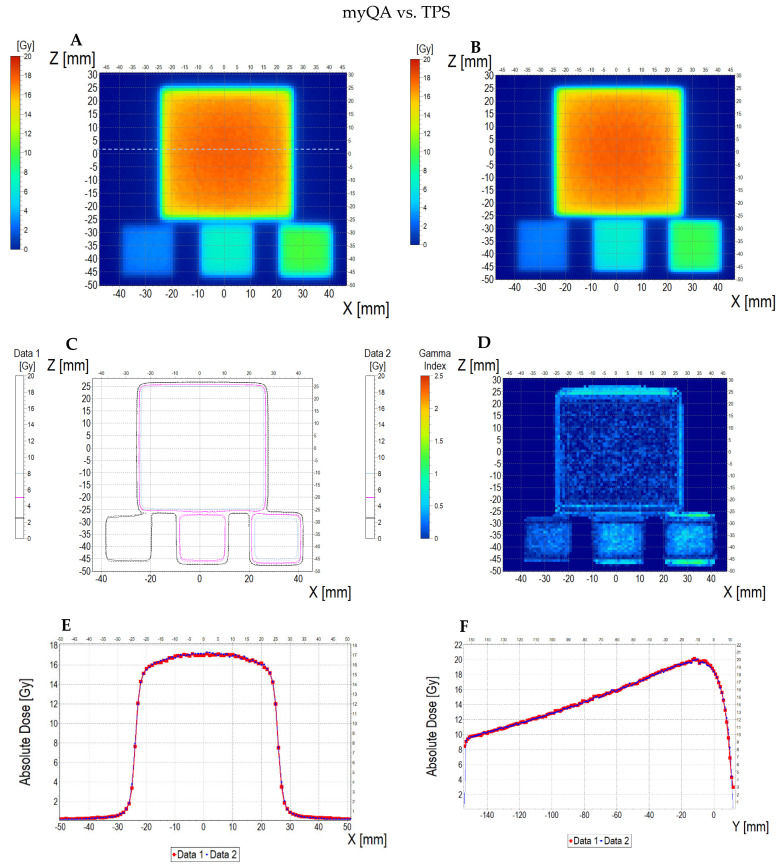

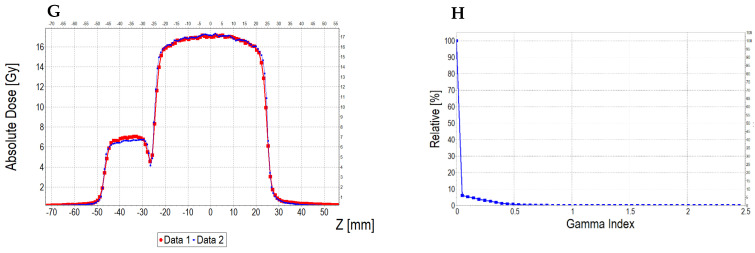
Comparison of dose distribution calculated with myQA iON/MC simulations and TPS. (**A**) and (**B**): ZX planes of dose distributions for myQA iON/MC simulations and TPS, respectively; (**C**): superimposition of isodoses; (**D**): gamma index (DD = 3%, DTA = 3 mm); (**E**–**G**): profiles along Z, Y, and Z axes (position of profiles is presented in (**A**) by the lilac dashed lines); (**H**): gamma index histogram.

The comparison of the dose distribution measured using a thin dosimeter (scan of the sample is presented in Supplementary Figure S6A) with the dose distribution calculated in the Eclipse Treatment Planning System (v. 16.1) and myQA iON software (v. 2.1.0) is presented in Figure 11 (dosimeter vs. myQA iON/MC simulations), Figure 12 (dosimeter vs. TPS), and Figure 13 (TPS vs. myQA iON/MC simulations). At a glance, it can be seen that the measured dose distribution in the area irradiated with the highest dose is non-uniform in contrast to the calculated distributions. The measured mean dose in the central area is 16.63 ± 1.20 Gy, while for TPS and myQA iON/MC simulations, it is 17.27 ± 0.50 Gy and 17.27 ± 0.60 Gy, respectively. These values amount to the percentage differences with respect to the measured dose equal to 3.85%. The mean dose values measured and calculated for the remaining irradiated areas are presented in Table 2. The comparison of the gamma index for the measured and calculated dose distributions is as follows: 25% of the pixels are above 1 for the dosimeter versus myQA iON/MC simulations, 27% of the pixels are above 1 for the dosimeter versus TPS, and 0% of the pixels are above 1 for the TPS versus myQA iON/MC simulations. It should be noted that gamma index histograms were also shown for regions constrained to better correspondences, where the homogeneity of the sample structure was suspected to be better than other regions (Figure 11D and inset in Figure 11H, and Figure 12D and inset in Figure 12H). For these regions, the percentage of pixels with gamma index values above 1 was acceptable. For the agreement between the measured and calculated dose distributions to be considered acceptable, the number of pixels with a gamma index below 1 must be within 0–5% [56], which means that the dose distribution calculations in TPS were successfully verified by myQA iON/MC simulations. However, the verification using the thin dosimeter was, in some ways, unsuccessful, apart from the confined regions mentioned earlier. Judging by the results in Figure 11D and Figure 1D, the inner left part of the larger square obtained satisfactory gamma index results, whereas the region on the right side has more pixels above 1. This strongly indicates non-uniformity of the manufactured dosimeter due to the adopted preliminary manufacturing technology and does not disqualify the system as a whole. The isodose superimposed, as presented in Figure 11C, outlines the square shapes of the irradiated regions and are mostly close to each other for both datasets; in some parts, they deviate from each other due to the poorer quality of images obtained for the dosimeter. The profiles along the X, Y, and Z axes for the calculated results in TPS and myQA iON/MC simulations are very similar. The only clear difference appeared along the Z axis in the range of −45–(−35) mm, where the dose value calculated in TPS is higher. Comparing the profiles obtained for the measured dose distribution with the profiles for the calculated dose distribution, a greater similarity is visible for the profiles along the Z axis. For the profiles along the X axis, a clear dose difference appears in the range of 0–25 mm. The verification of the treatment plan showed large differences between the measured and calculated dose distributions. The source of the differences may be the insufficient quality of the scanner used and the impact of structure of the foil covering the gel. In the measured dose distribution Figure 11A, vertical stripes are visible in the area irradiated with the highest dose (phenomenon described in Section 3.4). Their presence is also visible in the profile along the X axis, which is wavy within the range of the highest dose. In addition, the results may have been influenced by the non-uniform thickness of the gel layer and mentioned possible imperfections of the rigid foil sheets. In order for the thin dosimeter verifying the dose distribution on the skin to be used for verifying the treatment plan, it is necessary to improve the methods of manufacturing and scanning the dosimeter. Nevertheless, it is clear that such a thin dosimeter showed a great promise in radiotherapy applications for TPS verification purposes.

#### 3.6.3. Treatment Planning Verification: Fricke-XO-Gelatine (Thick Gel)

**Figure 14:**
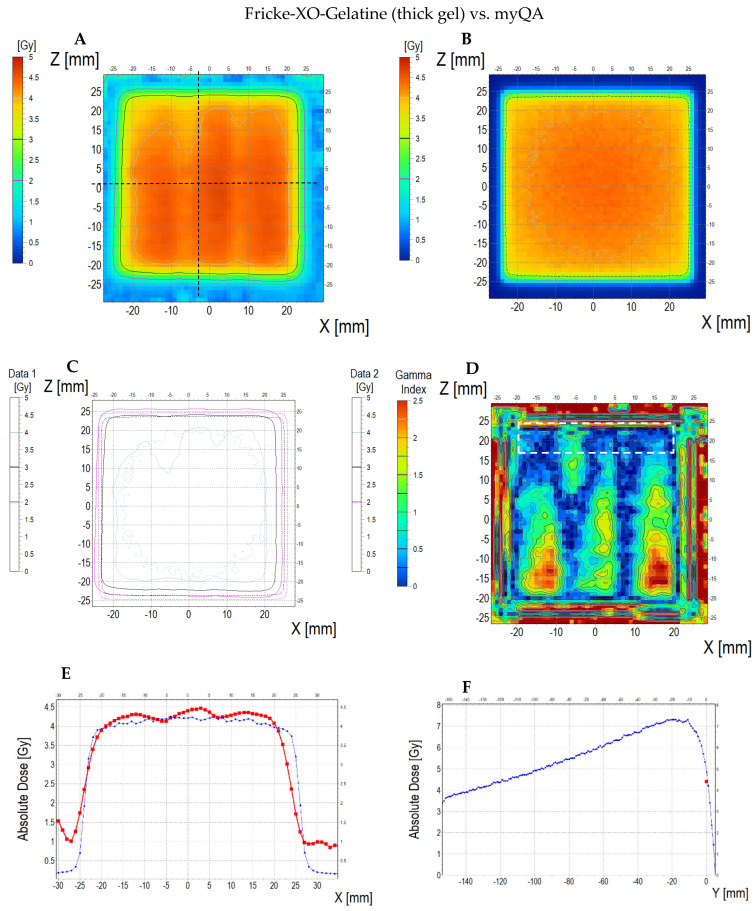

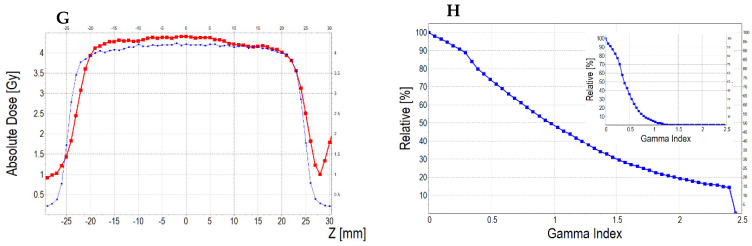
Comparison of dose distribution measured with Fricke-XO-Gelatine 2D dosimeter (thick gel) and calculated with myQA iON/MC simulations. (**A**) and (**B**): ZX planes of dose distributions for Fricke-XO-Gelatine and myQA iON/MC simulations, respectively; (**C**): superimposition of isodoses; (**D**): gamma index (DD = 3%, DTA = 3 mm); (**E**–**G**): profiles along Z, Y, and Z axes (position of profiles is presented in (**A**) by the black dashed lines); (**H**): gamma index histogram. Inset in (**H**): a gamma index histogram for a ROI indicated in (**D**) with the white dashed line; 4.4% pixels above gamma index = 1.

**Figure 15:**
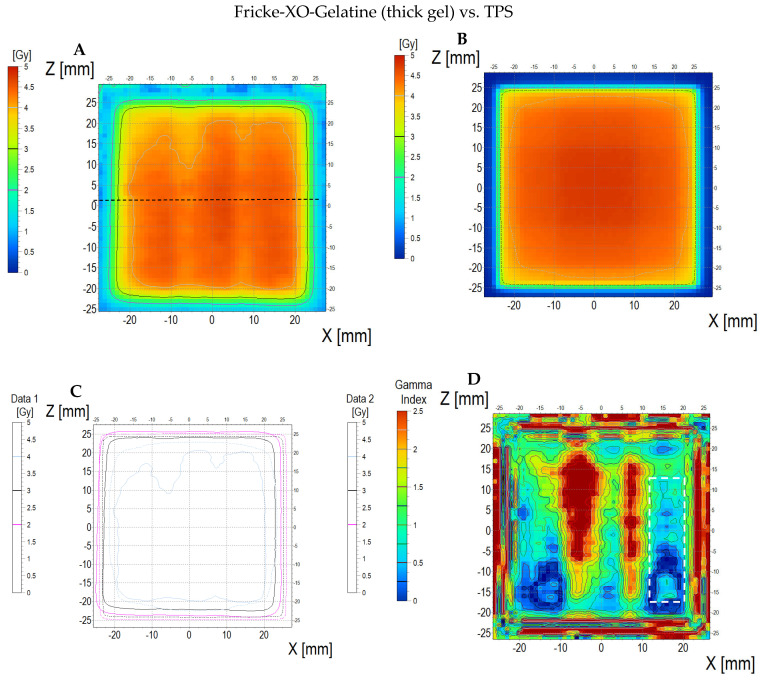

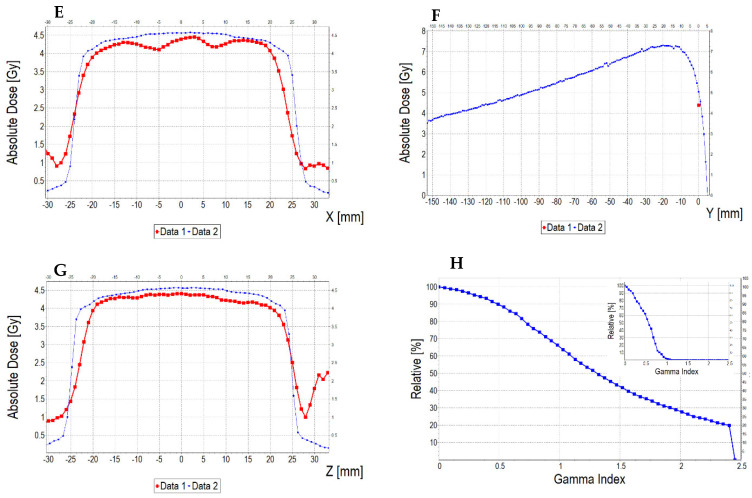
Comparison of dose distribution measured with Fricke-XO-Gelatine 2D dosimeter (thick gel) and calculated with TPS. (**A**) and (**B**): ZX planes of dose distributions for Fricke-XO-Gelatine and TPS, respectively; (**C**): superimposition of isodoses; (**D**): gamma index (DD = 3%, DTA = 3 mm); (**E**–**G**): profiles along Z, Y, and Z axes (position of profiles is presented in (**A**) by the black dashed lines); (**H**): gamma index histogram. Inset in (**H**): a gamma index histogram for a ROI indicated in (**D**) with the white dashed line; 1.8% pixels above gamma index = 1.

**Figure 16:**
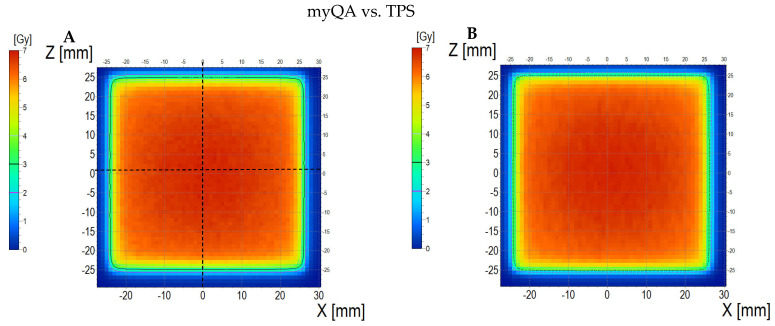

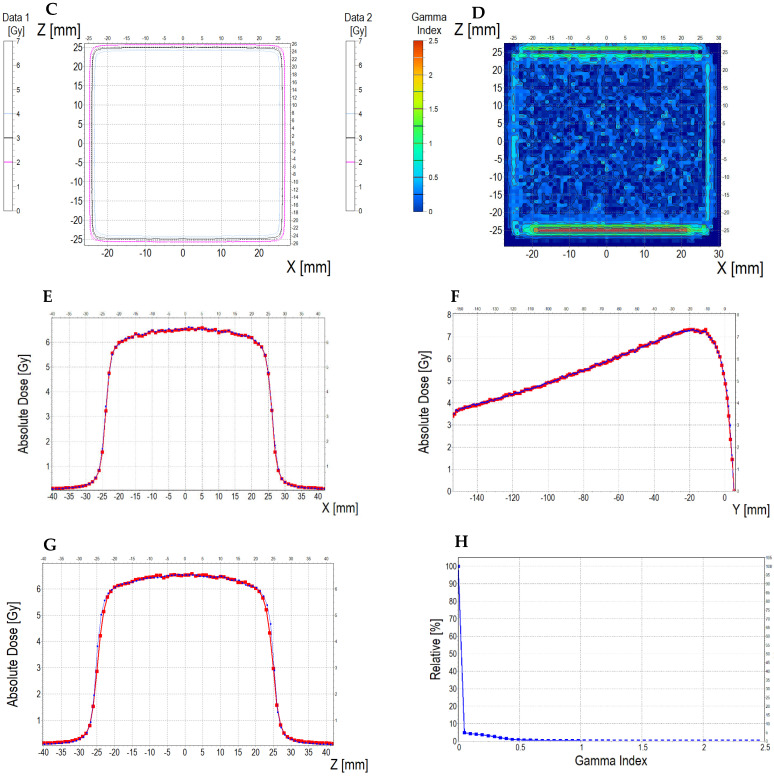
Comparison of dose distribution calculated with myQA iON/MC simulations and TPS. (**A**) and (**B**): ZX planes of dose distributions for myQA iON/MC simulations and TPS, respectively; (**C**): superimposition of isodoses; (**D**): gamma index (DD = 3%, DTA = 3 mm); (**E**–**G**): profiles along Z, Y, and Z axes (position of profiles is presented in (**A**) by the black dashed lines); (**H**): gamma index histogram.

The comparison of the dose distribution measured using a thick dosimeter (scan of the sample is presented in Supplementary Figure S6B) with the dose distribution calculated in the TPS and simulated with aid of the myQA iON software/MC simulations is presented in Figure 14 (dosimeter vs. myQA iON/MC simulations), Figure 15 (dosimeter vs. TPS), and Figure 16 (TPS vs. myQA iON/MC simulations). Similar to the thin dosimeter verification, the measured dose distribution is non-uniform and takes the shape of three wide bands of higher dose separated by narrow bands of lower dose. The measured mean dose in the irradiated area was 4.16 ± 0.20 Gy, while the doses calculated for TPS and myQA iON/MC simulations were 4.36 ± 0.10 Gy and 4.06 ± 0.10 Gy, respectively. The percentage difference between the measured mean dose and the calculated dose for TPS is −4.81%, which is within the required accuracy of ±5% [57,58]. The percentage difference between the mean doses calculated by myQA iON/MC simulations and TPS was −7.39%, which does not meet the required accuracy. Gamma index values exceed 1 for 50% of pixels comparing measured and calculated doses for TPS and for 65% of pixels comparing measured and calculated doses in myQA iON/MC simulations. For TPS vs. myQA iON/MC simulations, 0% of pixels had a gamma index value higher than 1. As in the case of the thin dosimeter, it should be noted that gamma index histograms were also shown for regions constrained to better correspondences, where the homogeneity of the sample structure was suspected to be better than other regions (Figure 14D and inset in Figure 14H and Figure 15D and inset in Figure 15H). For these regions, the percentage of pixels with gamma index values above 1 was acceptable. Similar to the thin dosimeter, verification of the treatment plan using the thick dosimeter was problematic in some aspects. While the mean measured and calculated dose values are similar, the way they are distributed differs significantly. The resulting differences are caused by the stripes visible in the scans. The presence of stripes is also visible in the profiles plotted along the X axis (across the stripes). The obtained profiles are wavy and differ in shape from the profiles obtained in TPS and myQA iON/MC simulations. The profiles along the Z axis (along the stripes) for the measured and calculated dose distributions are similar in shape. The presence of bands may be caused by poor quality of scanning, scanner quality, and suboptimal quality of the foils covering the dosimeter on both sides, or by other issues unrecognized in this study. However, there is most likely an association between the currently adopted manufacturing method of the dosimeters and scanning issues. Further research is needed to optimize dosimeter performance and scanning despite the fact that these preliminary results prove the potential of the dosimetry system developed.

## 4. Conclusions

The aim of this study was to investigate the effect of sorbitol addition on the mechanical and thermal properties of the Fricke-XO-Gelatin dosimeter, examine its chemical stability and dose response characteristics, and to present the concept of using two-dimensional gel dosimeters as tools enabling their simultaneous use as a bolus and *in vivo* dosimeter while additionally enabling the performance of a medical accelerator test.

The addition of sorbitol improved the mechanical and thermal parameters of gelatin; however, at the concentration used, it was not able to completely mitigate the effect of the acidic Fricke solution, the presence of which leads to a decrease in the sol–gel transition temperature of the dosimeter and a weakening of its mechanical strength. The dosimeter maintains chemical stability, enabling its use in dosimetric applications, for at least six weeks. The dose–response characteristics pointed to a dynamic dose response of the dosimeter of ~20 Gy and a linear dose response of ~12.5 Gy. The two-dimensional Fricke-XO-Gelatin dosimeter with the addition of sorbitol proved to be a suitable tool for conducting a test of the coincidence of the radiation and mechanical isocenter of the accelerator. The ability to manufacture dosimeters of different thicknesses and sizes, as well as the lack of angular dependencies, allows this dosimeter to be used as a bolus and an *in vivo* dosimeter. The verification of the dose distribution calculated by TPS and Monte Carlo simulations using thick (bolus) and thin dosimeters encountered some adversarial issues related to both scanning and the gel manufacturing quality, which affected some regions of the gel (non-uniformity in dose distribution) that in turn reflected poor gamma index values.

The conducted studies have shown both the potential of the presented 2D dosimetry system (dosimeter with flatbed scanner and dedicated software) and the need for further work on obtaining improved two-dimensional gel dosimeters for use in ensuring the quality of radiotherapy. Potential paths for further development include the following: (i) optimization of the composition and method of manufacturing the dosimeter, (ii) searching for other matrix materials with better mechanical properties, and (iii) using other radiation-sensitive systems that are potentially less irritating in direct contact with the patient’s skin.

## Figures and Tables

**Table 1:** Comparison of Fricke-XO-Gelatin with sorbitol with other deformable dosimeters.

No	Type of Dosimeter	Basic Reaction	Composition	Sensitivity	Linear Range	Ref
1	Fricke-XO-Gelatin with sorbitol (bolus)	Oxidation of Fe^2+^ ions	7.96% gelatin, 50 mM H_2_SO_4_, 0.5 mM FAS, 0.165 mM XO, 23% sorbitol	5.7579 Gy^−1^	12.5 Gy	This work
2	Fricke-XO-Gelatin with sorbitol (thin)	Oxidation of Fe^2+^ ions	7.96% gelatin, 50 mM H_2_SO_4_, 0.5 mM FAS, 0.165 mM XO, 23% sorbitol	4.9378 Gy^−1^	12.5 Gy	This work
3	DEFGEL	Polymerization of acrylamide monomers	6% gelatin, 3% *N*,*N*’-methylene-bis-acrylamid, monomer 3% acrylamid monomer, 5 mM bis [tetrakis (hydroksymethyl-phosphonium)] sulfate, 0.01 mM hydroquinone	0.02 cm^−1^ Gy^−1^	18.89 Gy (Maximum dose used in the study)	[14]
4	Polymer gel dosimeter enclosed using LDPE wraps	Polymerization of methacrylic acid monomers	8% gelatin, 5% methacrylic acid, 50 mM bis [tetrakis (hydroksymethyl-phosphonium)]	0.86 s^−1^ Gy^−1^	4 Gy (Maximum dose used in the study)	[15]
5	LMG-silicone	Oxidation of LMG to malachite green	Dow Corning SYLGARD^®^ 184 Silicone Elastomer Kit (the ratio of curing agent to silicone elastomer was 1:10), 0.4 mM LMG, 200 mM chloroform	0.012 cm^−1^ Gy^−1^	60 Gy (Maximum dose used in the study)	[19]

**Table 2:** Comparison of doses for Fricke-XO-Gelatin with sorbitol thin and thick gel dosimeters with myQA iON/MC simulations (abbreviated to myQA in this table) and TPS for a ROI of 40 × 40 mm^2^ and 15×14 mm^2^ (smaller regions are shown in Figure 11, Figure 12 and Figure 13), located in the middle of the irradiated square regions (image in plane resolution: 1 × 1 mm^2^).

No.	Parameter	For Thin Fricke-XO-Gelatin Gel (Four Square Regions Irradiated)						For Thick Fricke-XO-Gelatin Gel (One Square Region Irradiated)					
		Fricke-XO-Gelatin vs. myQA		Fricke-XO-Gelatin vs. TPS		myQA vs. TPS		Fricke-XO-Gelatin vs. myQA		Fricke-XO-Gelatin vs. TPS		myQA vs. TPS	
		Fricke-XO-Gelatin (1)	myQA (2)	Fricke-XO-Gelatin (1)	TPS (2)	myQA (1)	TPS (2)	Fricke-XO-Gelatin (1)	myQA (2)	Fricke-XO-Gelatin (1)	TPS (2)	myQA (1)	TPS (2)
1	Minimum dose [Gy]	12.06	15.23	12.06	15.42	15.23	15.42	3.54	3.67	3.54	3.86	3.67	3.86
		2.95	2.54	2.95	2.63	2.54	2.63						
		4.50	4.82	4.50	5.07	4.82	5.07						
		7.04	6.94	7.04	7.36	6.94	7.36						
2	Maximum dose [Gy]	18.60	18.27	18.60	18.14	18.27	18.14	4.48	4.27	4.48	4.57	4.27	4.57
		4.25	3.83	4.25	3.61	3.83	3.61						
		7.42	7.45	7.42	7.10	7.45	7.10						
		10.76	10.81	10.76	10.25	10.81	10.25						
3	Mean dose [Gy]	16.63	17.27	16.63	17.27	17.27	17.27	4.16	4.06	4.16	4.36	4.06	4.36
		3.92	3.55	3.92	3.41	3.55	3.41						
		6.77	6.95	6.77	6.73	6.95	6.73						
		9.69	9.99	9.69	9.74	9.99	9.74						
4	STD dose [Gy]	1.2	0.6	1.2	0.5	0.6	0.5	0.2	0.1	0.2	0.1	0.1	0.1
		0.3	0.2	0.3	0.2	0.2	0.2						
		0.6	0.5	0.6	0.4	0.5	0.4						
		0.9	0.8	0.9	0.6	0.8	0.6						
5	Mean dose difference [%] = 100 – (2) × 100/(1)	−3.85		−3.85		0.00		2.40		−4.81		−7.39	
		9.44		13.01		3.94							
		2.66		0.59		3.17							
		3.10		0.52		2.50							

## Data Availability

The data supporting the reported results are not stored in any publicly archived datasets. The readers can contact the corresponding author for any further clarification of the results obtained.

## Supplementary Materials

The following supporting information can be downloaded at: https://www.mdpi.com/article/10.3390/ma18133135/s1.

## References

[1] Baldock C., De Deene Y., Doran S., Ibbott G., Jirasek A., Lepage M., McAuley K.B., Oldham M., Schreiner L.J. (2010). Polymer gel dosimetry. Phys. Med. Biol..

[2] De Deene Y. (2022). Radiation dosimetry by use of radiosensitive hydrogels and polymers: Mechanisms, state-of-the-art and perspective from 3D to 4D. Gels.

[3] Watanabe Y., Warmington L., Gopishankar N. (2017). Three-dimensional radiation dosimetry using polymer gel and solid radiochromic polymer: From basics to clinical applications. World J. Radiol..

[4] Macchione M.A., Páez S.L., Strumia M.C., Valente M., Mattea F. (2022). Chemical overview of gel dosimetry systems: A comprehensive review. Gels.

[5] Dorsh S., Mann P., Elter A., Runz A., Klüter S., Karger C.P. (2019). Polymer gel-based measurements of the isocenter accuracy in an MR-LINAC. J. Phys. Conf. Ser..

[6] Dorsh S., Mann P., Lang C., Hearing P., Runz A., Karger C.P. (2018). Feasibility of polymer gel-based measurements of radiation isocenter accuracy in magnetic fields. Phys. Med. Biol..

[7] Kozicki M., Maras P. (2023). On the measurement of radiation isocenter for medical accelerators using 3D polymer gel dosimetry. Introduction, application, and good practices. Measurement.

[8] Kim J.H., Kim B., Shin W.G., Son J., Choi C.H., Park J.M., Hwang U.J., Jin K., Jung S. (2022). 3D star shot analysis using MAGAT gel dosimeter for integrated imaging and radiation isocenter verification of MR-Linac system. J. Appl. Clin. Med. Phys..

[9] Piotrowski M., Maras P., Kozicki M. (2024). On the use of the Fricke-Pluronic F-127 gel dosimeter for radiation isocenter testing of a medical linear accelerator. Materials.

[10] Kozicki M., Maras P. (2024). An optical reusable 2D radiochromic gel-based system for ionising radiation measurements in radiotherapy. Molecules.

[11] Aon E., Brunetto M., Sansogne R., Castellano G., Valente M. (2008). Novel Method Based on Fricke Gel Dosimeters for Dose Verification in IMRT Techniques. Proceedings of the 12th International Congress of the International Radiation Protection Association (IRPA 12).

[12] Valente M., Aon E., Brunetto M., Castellano G., Gallivanone F., Gambarini G. (2007). Gel Dosimetry Measurements and Monte Carlo Modeling for External Radiotherapy Photon Beams: Comparison with a Treatment Planning System Dose Distribution. Nucl. Instrum. Methods Phys. Res. A.

[13] Chow J.C.L., Ruda H.E. (2025). In Vivo Dosimetry in Radiotherapy: Techniques, Applications, and Future Directions. Encyclopedia.

[14] Yeo U.J., Taylor L., Dunn L., Kron T., Smith R.L., Franich R.D. (2012). A Novel Methodology for 3D Deformable Dosimetry. Med. Phys..

[15] Niu C.J., Foltz W.D., Velec M., Moseley J.L., Al-Mayah A. (2012). A Novel Technique to Enable Experimental Validation of Deformable Dose Accumulation. Med. Phys..

[16] Juang T., Das S., Adamovics J., Benning R., Oldham M. (2013). On the Need for Comprehensive Validation of Deformable Image Registration, Investigated with a Novel 3D Deformable Dosimeter. Int. J. Radiat. Oncol. Biol. Phys..

[17] Kaplan L.P., Høye E.M., Balling P., Muren L.P., Petersen J.B.B., Poulsen P.R., Yates E.S., Skyt P.S. (2017). Determining the Mechanical Properties of a Radiochromic Silicone-Based 3D Dosimeter. Phys. Med. Biol..

[18] De Deene Y., Skyt P.S., Hill R., Booth J.T. (2015). FlexyDos3D: A Deformable Anthropomorphic 3D Radiation Dosimeter: Radiation Properties. Phys. Med. Biol..

[19] Høye E.M., Skyt P.S., Yates E.S., Muren L.P., Petersen J.B.B., Balling P. (2015). A New Dosimeter Formulation for Deformable 3D Dose Verification. J. Phys. Conf. Ser..

[20] Fricke H., Morse S. (1927). The Chemical Action of Roentgen Rays on Dilute Ferrous Sulphate Solutions as a Measure of Radiation Dose. Am. J. Roentgenol. Radium Ther. Nucl. Med..

[21] Gore J.C., Kang Y.S., Shultz R.J. (1984). Measurement of radiation dose distributions by nuclear magnetic resonance (NMR) imaging. Phys. Med. Biol..

[22] Bero M.A., Gilboy W.B., Glover P.M. (2001). Radiochromic gel dosemeter for three-dimensional dosimetry. Radiat. Phys. Chem..

[23] Baldock C., Harris P.J., Piercy A.R., Healy B. (2001). Experimental determination of the diffusion coefficient in two dimensions in ferrous sulphate gels using the finite element method. Australas. Phys. Eng. Sci. Med..

[24] Kelly R.G., Jordan K.J., Battista J.J. (1998). Optical CT reconstruction of 3D dose distributions using the ferrous–benzoic–xylenol (FBX) gel dosimeter. Med. Phys..

[25] Pappas E.P., Peppa V., Hourdakis C.J., Karaiskos P., Papagiannis P. (2018). On the use of a novel Ferrous Xylenol-orange gelatin dosimeter for HDR brachytherapy commissioning and quality assurance testing. Phys. Med..

[26] Jordan K., Battista J. (2004). Dose response of ferrous-xylenol orange gels: The effects of gel substrate, gelation time and dose fractionation. J. Phys. Conf. Ser..

[27] Magugliani G., Liosi G.M., Anderoli S., De Crescenzo S., Mossini E., Macerata E., Mariani M. (2020). Characterization of Fricke-gelatin dosimeters for intraoperative Radiation Therapy dosimetry. Radiat. Phys. Chem..

[28] Olsson L.E., Appleby A., Sommer I. (1991). A new dosimeter based on ferrous sulphate solution and agarose gel. Appl. Radiat. Isot..

[29] Chu K.C., Jordan K.J., Battista J.J., Van Dyk J., Rutt B.K. (2000). Polyvinyl alcohol–Fricke hydrogel and cryogel: Two new gel dosimetry systems with low Fe3+ diffusion. Phys. Med. Biol..

[30] Rabaeh K.A., Eyadeh M.M., Hailat T.F., Madas B.G., Aldweri F.M., Almomani A.M., Awad S.I. (2021). Improvement on the performance of chemically cross-linked Fricke methylthymol-blue radiochromic gel dosimeter by addition of dimethyl sulfoxide. Radiat. Meas..

[31] d’Errico F., Lazzeri L., Dondi D., Mariani M., Marrale M., Souza S.O., Gambarini S. (2017). Novel GTA-PVA Fricke gels for three-dimensional dose mapping in radiotherapy. Radiat. Meas..

[32] Dudek M., Piotrowski M., Maras P., Jaszczak M., Kozicki M. (2021). Anisotropic diffusion of Fe ions in Fricke-XO-Pluronic F-127 and Fricke-XO-Gelatine 3D radiotherapy dosimeters. Phys. Med. Biol..

[33] Piotrowski M., Maras P., Kadłubowski S., Kozicki M. (2022). Study of the Optimal Composition and Storage Conditions of the Fricke–XO–Pluronic F–127 Radiochromic Dosimeter. Materials.

[34] Piotrowski M., Maras P., Wach R., Kadłubowski S., Kozicki M. (2022). Impact of Salt on Thermal Stability and Dose Response of the Fricke-XO-Pluronic F-127 3D Radiotherapy Dosimeter. Materials.

[35] Eyadeh M.M., Rabaeh K.A., Alshomali L.S., Diamond K.R., Oglat A.A. (2024). Evaluation of a novel physically cross-linked Fricke-xylenol orange-polyvinyl alcohol radio-chromic gel dosimeter for radiotherapy. Radiat. Meas..

[36] Jordan K., Sekimoto M. (2010). Effects of adding glycerol and sucrose to ferrous xylenol orange hydrogel. J. Phys. Conf. Ser..

[37] Piotrowski M., Maras P., Kozicki M. (2024). Sorbitol to reduce Fe diffusion in a Fricke gel dosimeter and enhance its resistance to elongation. J. Phys. Conf. Ser..

[38] Wang R., Hartel R.W. (2022). Citric acid and heating on gelatin hydrolysis and gelation in confectionery gels. Food Hydrocoll..

[39] Martucci J.F., Accareddu A.E.M., Ruseckaite R.A. (2012). Preparation and characterization of plasticized gelatin films cross-linked with low concentrations of Glutaraldehyde. J. Mater. Sci..

[40] Dai H., Li X., Du J., Ma L., Yu Y., Zhou H., Guo T., Zhang Y. (2020). Effect of interaction between sorbitol and gelatin on gelatin properties and its mechanism under different citric acid concentrations. Food Hydrocoll..

[41] Lu Y., Song J., Yao X., An M., Shi Q., Huang X. (2021). 3D Printing Polymer-based Bolus Used for Radiotherapy. Int. J. Bioprint..

[42] Chen X., Wang W., Huang X., Diao W., Xu C., Jia L., Li H., Li B., Jiang X. (2024). Physical and Dosimetric Characterization of Silicone Rubber Bolus for Head Photon-Beam Radiotherapy. Technol. Cancer Res. Treat..

[43] Sianturi H.A., Ginting J., Azhari, Rianna M., Marbun R.G.A., Sinaga A.P., Siregar R.Y.M., Sidaruk L., Silalahi M., Tampubolon H. (2024). Enhancing Radiotherapy Bolus Characteristics through Comparative Analysis of Added Sawdust and Bagasse Powder: A Comprehensive Study of Physical and Mechanical Properties. J. Phys. Conf. Ser..

[44] Gül O.V. (2023). Investigation of Surface Dose Accuracy of Two Dose Calculation Algorithms Using Thermoluminescent Dosimeters. GU J. Sci..

[45] Sheykholeslami N., Parwaie W., Farzin M., Vaezzadeh V., Esfahani M., Geraily G. (2023). An Investigation into the Surface Dose Using Eclipse Treatment Planning System and Film Dosimetry for Treatment of Breast Cancer. Front. Biomed. Technol..

[46] Sigamani A., Nambiraj A., Yadav G., Giribabu A., Srinivasan K., Gurusamy V., Raman K., Karunakaran K., Thiyagarajan R. (2016). Surface Dose Measurements and Comparison of Unflattened and Flattened Photon Beams. J. Med. Phys..

[47] Dias A.G., Pinto D.F.S., Borges M.F., Pereira M.H., Santos J.A.M., Cunha L.T., Lencart J. (2019). Optimization of Skin Dose Using In-Vivo MOSFET Dose Measurements in Bolus/Non-Bolus Fraction Ratio: A VMAT and a 3DCRT Study. J. Appl. Clin. Med. Phys..

[48] Kozicki M., Jaszczak M., Maras P., Kadłubowski S. (2023). Measurement of the Radiation Dose and Radiation Isocenter of the TrueBeam Accelerator Using 3D Polymer Gel Dosimeters from the VIPAR Family with Different Chemical History. Measurement.

[49] Maras P., Kozicki M. (2022). Fast Isocenter Determination Using 3D Polymer Gel Dosimetry with Kilovoltage Cone-Beam CT Reading and the PolyGeVero-CT Software Package for Linac Quality Assurance in Radiotherapy. Materials.

[50] Kozicki M., Maras P., Karwowski A.C. (2014). Software for 3D Radiotherapy Dosimetry. Validation. Phys. Med. Biol..

[51] Martucci J.F., Ruseckaite R.A. (2009). Tensile Properties, Barrier Properties, and Biodegradation in Soil of Compression—Molded Gelatin-Dialdehyde Starch Films. J. Appl. Polym. Sci..

[52] Rivero S., Garcia M.A., Pinotti A. (2010). Correlations between Structural, Barrier, Thermal and Mechanical Properties of Plasticized Gelatin Films. Innov. Food Sci. Emerg. Technol..

[53] Kwiatos K., Maras P., Kadłubowski S., Stempień Z., Dudek M., Kozicki M. (2018). Tetrazolium Salts–Pluronic F-127 Gels for 3D Radiotherapy Dosimetry. Phys. Med. Biol..

[54] Bero M.A., Abukassem I. (2009). Detection of Ultraviolet Radiation Using Tissue Equivalent Radiochromic Gel Materials. J. Phys. Conf. Ser..

[55] Klein E.E., Hanley J., Bayouth J., Yin F.F., Simon W., Dresser S., Serago C., Aguirre F., Ma L., Arjomandy B. (2009). Task Group 142 Report: Quality Assurance of Medical Accelerators. Med. Phys..

[56] Stock M., Kroupa B., Georg D. (2005). Interpretation and Evaluation of the γ Index and the γ Index Angle for the Verification of IMRT Hybrid Plans. Phys. Med. Biol..

[57] Thwaites D. (2013). Accuracy Required and Achievable in Radiotherapy Dosimetry: Have Modern Technology and Techniques Changed Our Views?. J. Phys. Conf. Ser..

[58] International Atomic Energy Agency (2013). Development of Procedures for In Vivo Dosimetry in Radiotherapy.

