# Supplementary material for: Deformable Fricke-XO-Gelatin Radiochromic Dosimeter of Ionizing Radiation and Its Applications in Quality Assurance Tests for Radiation Therapy
Source: Materials (Basel). 2025 Jul 2;18(13):3135. doi: 10.3390/ma18133135 (PMC12250857; doi:10.3390/ma18133135)
Supplement: Supplementary file 1 [file materials-18-03135-s001.zip › materials-3706365-supplementary.pdf]

Supplementary material

Deformable Fricke-XO-Gelatin Radiochromic Dosimeter of Ionizing Radiation and Its Applications in Quality Assurance Tests for Radiation Therapy

Michał Piotrowski [1], Piotr Maras [2], Zbigniew Stempień [3], Radosław Wach [4] and Marek Kozicki [1,5,*]

[1] Department of Mechanical Engineering, Informatics and Chemistry of Polymer Materials, Faculty of Materials Technologies and Textile Design, Lodz University of Technology, 90-543 Lodz, Poland; michal.piotrowski@dokt.p.lodz.pl
[2] Department of Radiotherapy Planning, Copernicus Hospital, 93-513 Lodz, Poland; p.maras@kopernik.lodz.pl
[3] Institute of Textile Architecture, Faculty of Materials Technologies and Textile Design, University of Technology, 90-543 Lodz, Poland; zbigniew.stempien@p.lodz.pl
[4] Institute of Applied Radiation Chemistry, Chemistry Faculty, Lodz University of Technology, 93-590 Lodz, Poland; radoslaw.wach@p.lodz.pl
[5] GeVero Co., 90-980 Lodz, Poland

* Correspondence: marek.kozicki@p.lodz.pl

Abstract

This work presents a Fricke radiochromic gel dosimeter with xylenol orange (XO) and a gelatin matrix modified with sorbitol. The dosimeter, combined with 2D scanning using a flatbed scanner and data processing using dedicated software packages, creates a radiotherapy dosimetry measurement system. The dosimeter reacts to ionizing radiation by changing color as a result of the formation of complexes of Fe^{3+} and XO molecules. It was characterized in terms of thermal and chemical stability and mechanical properties. The presence of sorbitol improved the mechanical and thermal properties of the dosimeter. The dosimeter maintains chemical stability, enabling its use in dosimetric applications, for at least six weeks. The dose–response characteristics of the dosimeter are discussed and indicate a dynamic dose–response of the dosimeter (up to saturation) of about 20 Gy and a linear dose–response of about 12.5 Gy. The following applications of the dosimeter are discussed: (i) as a 2D dosimeter in a plastic container for performing a coincidence test of radiation and mechanical isocenters of a medical accelerator, and (ii) for *in vivo* dosimetry as a 2D dosimeter alone and simultaneously as a bolus and a 2D dosimeter. Research has shown that the dosimeter has promise in many applications.

Keywords: Fricke gel dosimeter; radiotherapy; 2D bolus dosimeter; flexible dosimeter; *in vivo* dosimetry

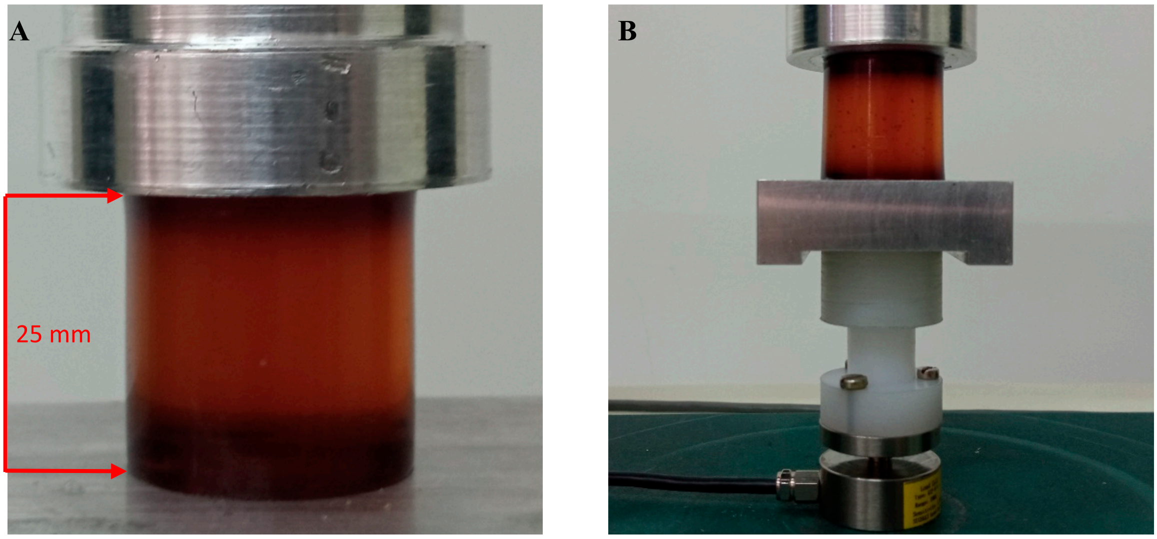

Supplementary Figure S1. Cylindrical sample of Fricke-XO-Gelatine with sorbitol with a height of 25 mm and a base diameter of 25 mm placed on an aluminum table and compressed at a speed of 10 mm/min with an aluminum pin with a diameter of 50 mm (**A**) and on an aluminum table located on the force meter in the cyclic compression system (**B**).

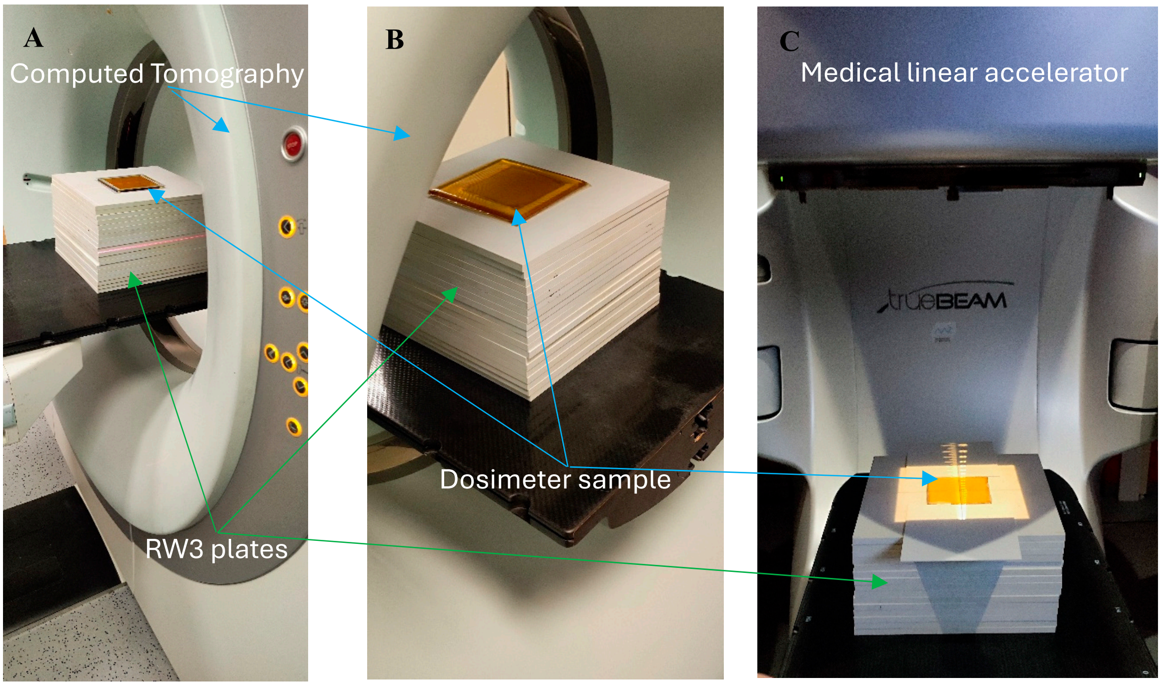

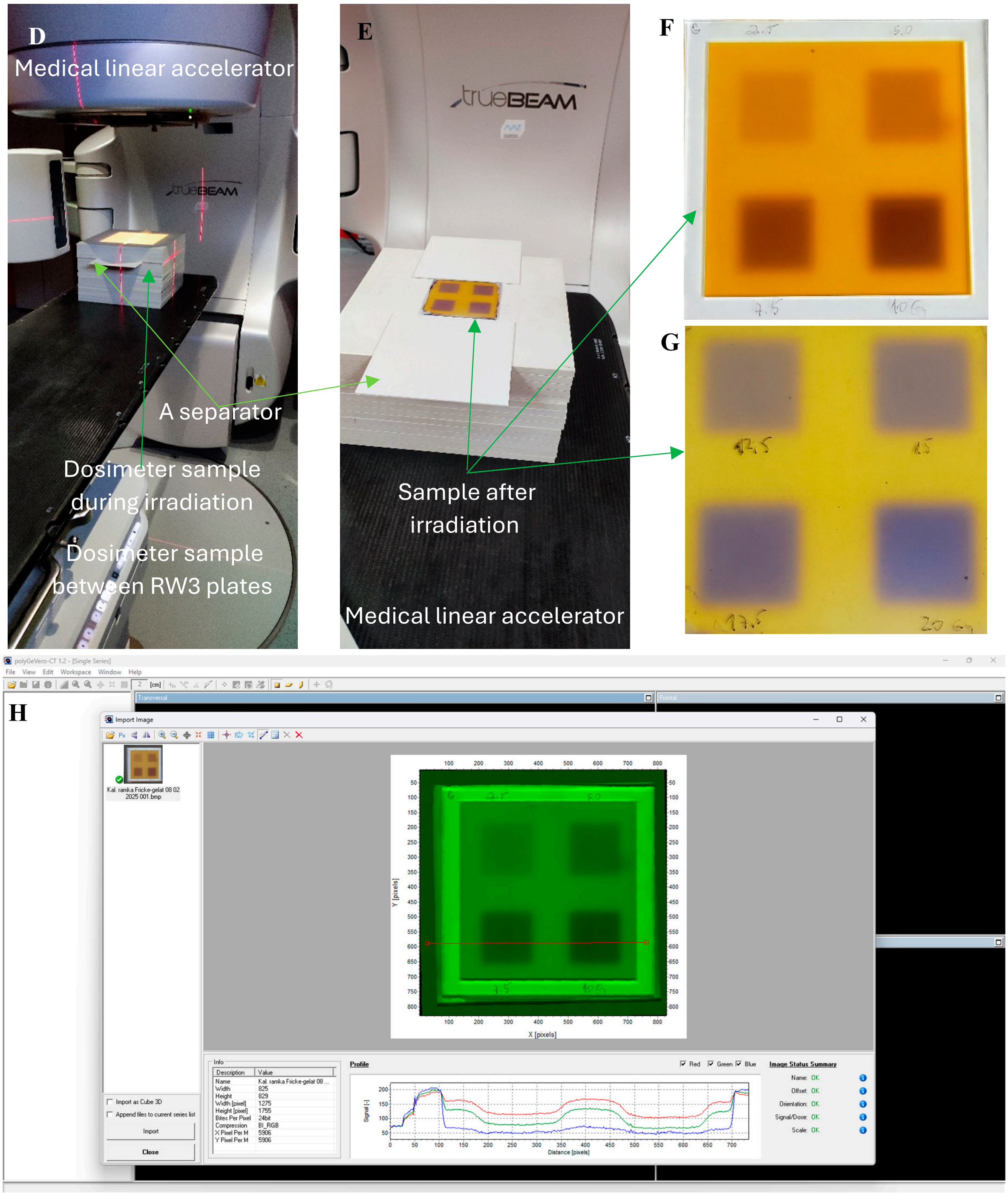
D
Medical linear accelerator
A separator
Dosimeter sample during irradiation
Dosimeter sample between RW3 plates
E
trueBEAM
Sample after irradiation
Medical linear accelerator
F
G
H
Import Image
X [pixels]
Y [pixels]
Profile
Distance [pixels]
Image Status Summary

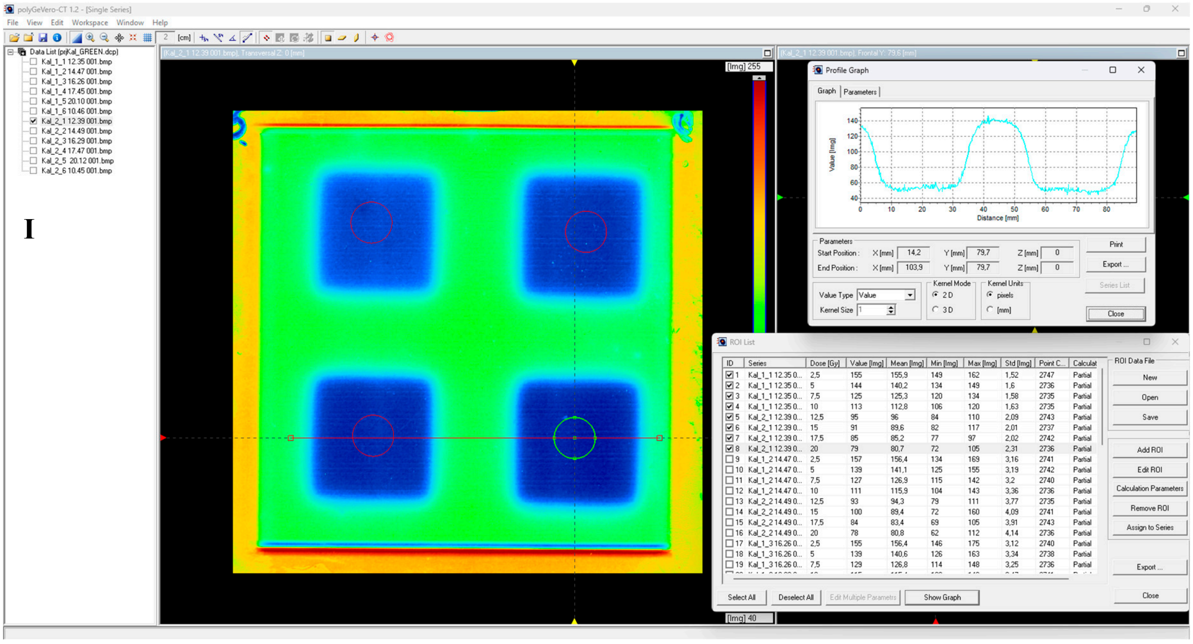
I
polyGeVero-CT 1.2 - [Single Series]
Profile Graph
ROI List

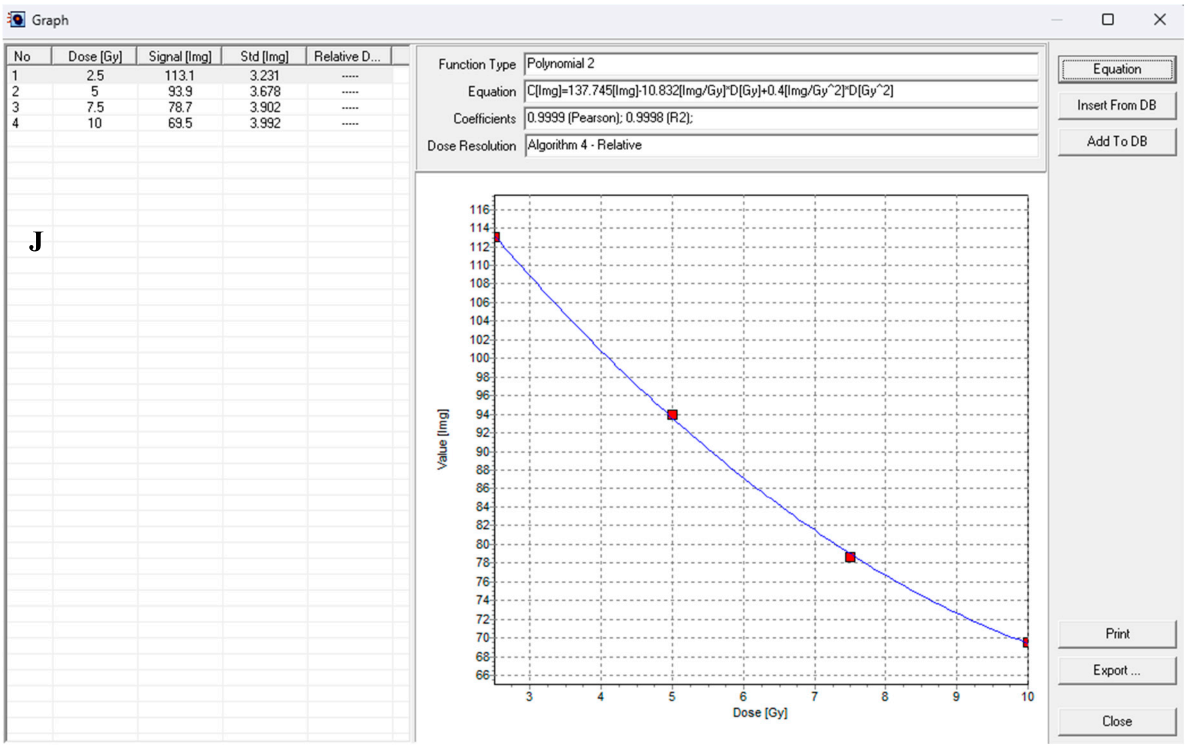
J
Graph
No
Dose [Gy]
Signal [Img]
Std [Img]
Relative D...
1 2.5 113.1 3.231 -----
2 5 93.9 3.678 -----
3 7.5 78.7 3.902 -----
4 10 69.5 3.992 -----
Function Type
Polynomial 2
Equation
C[Img]=137.745[Img]-10.832[Img/Gy]*D[Gy]+0.4[Img/Gy^2]*D[Gy^2]
Coefficients
0.9999 (Pearson); 0.9998 (R2);
Dose Resolution
Algorithm 4 - Relative
Equation
Insert From DB
Add To DB
Print
Export ...
Close
Value [Img]
Dose [Gy]

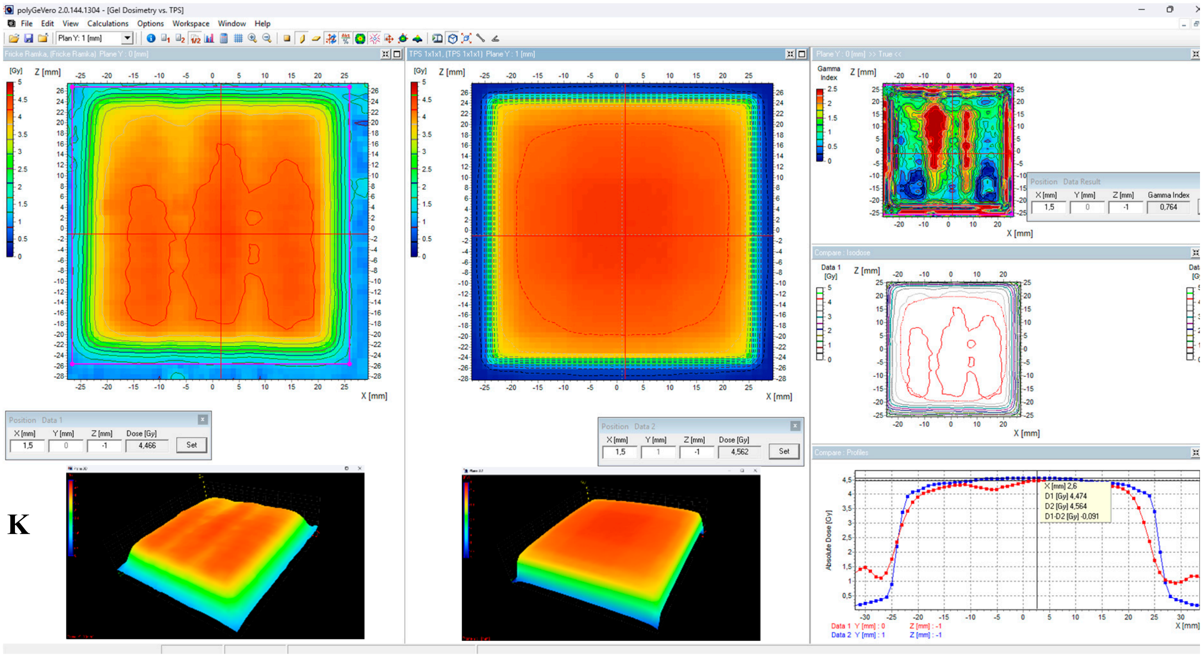

Supplementary Figure S2. Steps performed after sample preparation by irradiation, scanning, and data processing to calculate calibration and compare the measured dose distribution from the Fricke-XO-Gelatin with sorbitol dosimeter with the calculated or simulated dose distributions using TPS or myQA iON/MC simulations. A and B are photographs of the gel dosimeter sample during CT scanning. The scans are used in the treatment planning system to generate the gel dosimeter irradiation plan. C, D, and E are photographs of the gel dosimeter before, during, and after irradiation with a medical linear accelerator (TrueBeam, Varian, USA). In C, the gel dosimeter sample is visible placed on a stack of RW3 plates, and light is emitted to establish the irradiation field. In D, the dosimeter sample is visible between the RW3 plates; Several plates were placed on the sample and the sample was secured to prevent it from being crushed and damaged by the separator protruding from the plates. The source-to-surface distance is set to obtain the appropriate dose build-up. In E, a photograph of the dosimeter sample after irradiation is shown. The irradiated areas with darker color and square shapes are clearly visible. In F and G, there are scans of the dosimeter samples (an HP Scanjet G3010 flatbed scanner, Hewlett-Packard, USA, was used): a thick bolus dosimeter and a thin dosimeter, respectively. In H-K, there are screenshots of some work areas of the polyGeVero-CT (H-J) and polyGeVero (K) software packages (GeVero Co., Poland) with sample calculated results. In H, the work area is presented during image import after scanning the dosimeter sample with a flatbed scanner. In I, the work area is presented after image import and during calibration relation calculation; regions of interest (ROI) are selected, from which the average signal values are calculated. In J, the result of calibration calculation is shown. Such calibration is used to convert the signal value of the gel dosimeter verification sample to 2D dose distribution. The 2D dose distribution is then exported to the polyGeVero software package. In K, sample 2D dose distributions are transferred to the polyGeVero workspace: the 2D dose distribution measured using Fricke-XO gelatin with sorbitol (left) and the 2D dose distribution calculated using TPS. Additionally, a gamma index map as well as isodoses for both data sets, profiles along the X-axis for both data sets, and views of the 2D dose distribution using the 3D view option for both datasets are visible.

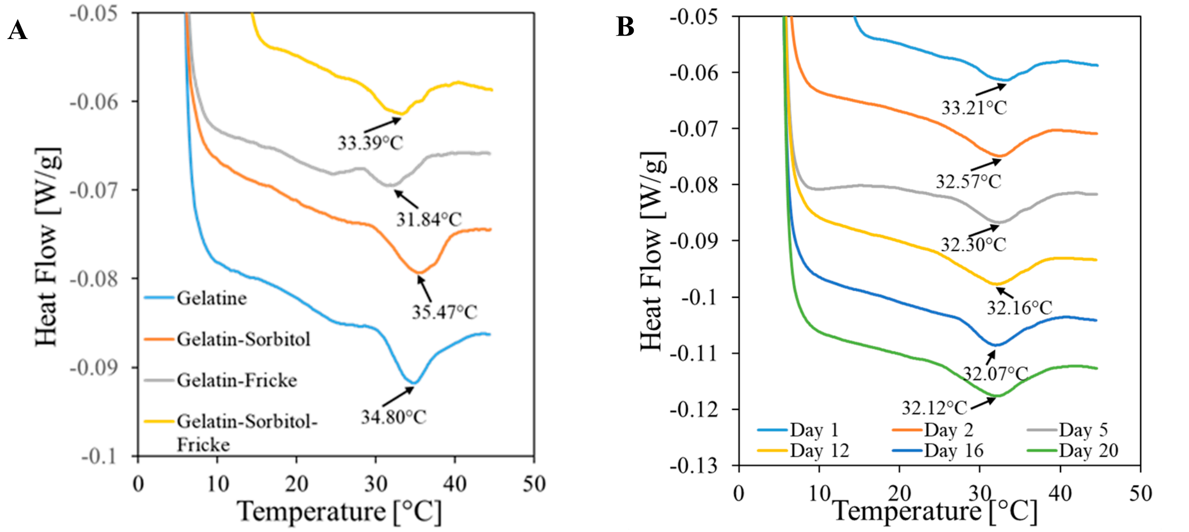

Supplementary Figure S3. **A**: Thermograms obtained for the first heating of 8% gelatin samples, 7.96% gelatin with Fricke, 6% gelatin with Fricke and 23% sorbitol, 5.99% gelatin with 23% sorbitol. The concentrations of the Fricke solution components in the samples were 50 mM H_2SO_4, 0.5 mM FAS, 0.165 mM XO. Measurements were performed 4 h after preparation. **B**: Thermograms for the first heating of gelatin with sorbitol and Fricke samples obtained at the following times from sample preparation: 1, 2, 5, 12, 16, and 20 days.

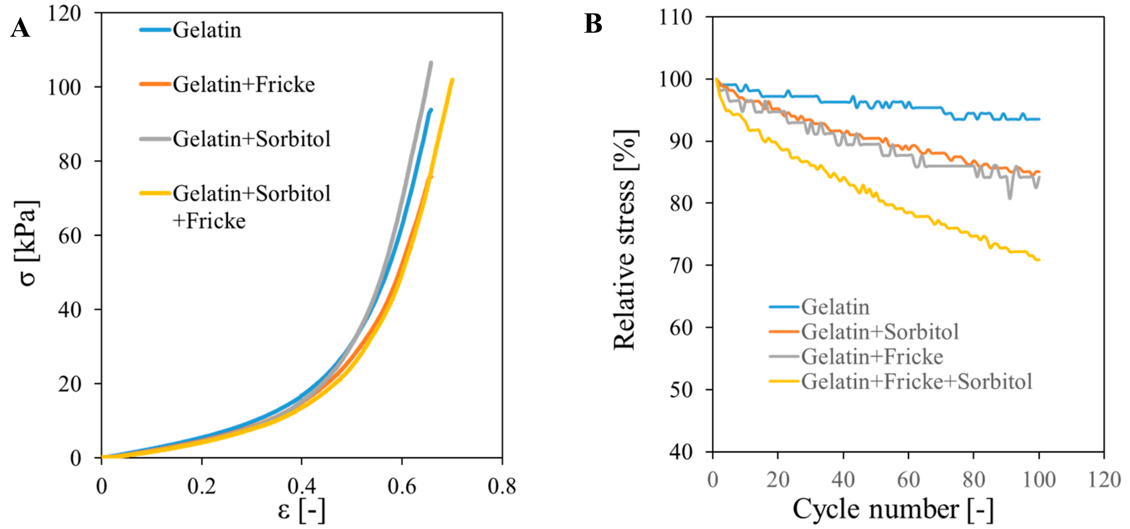

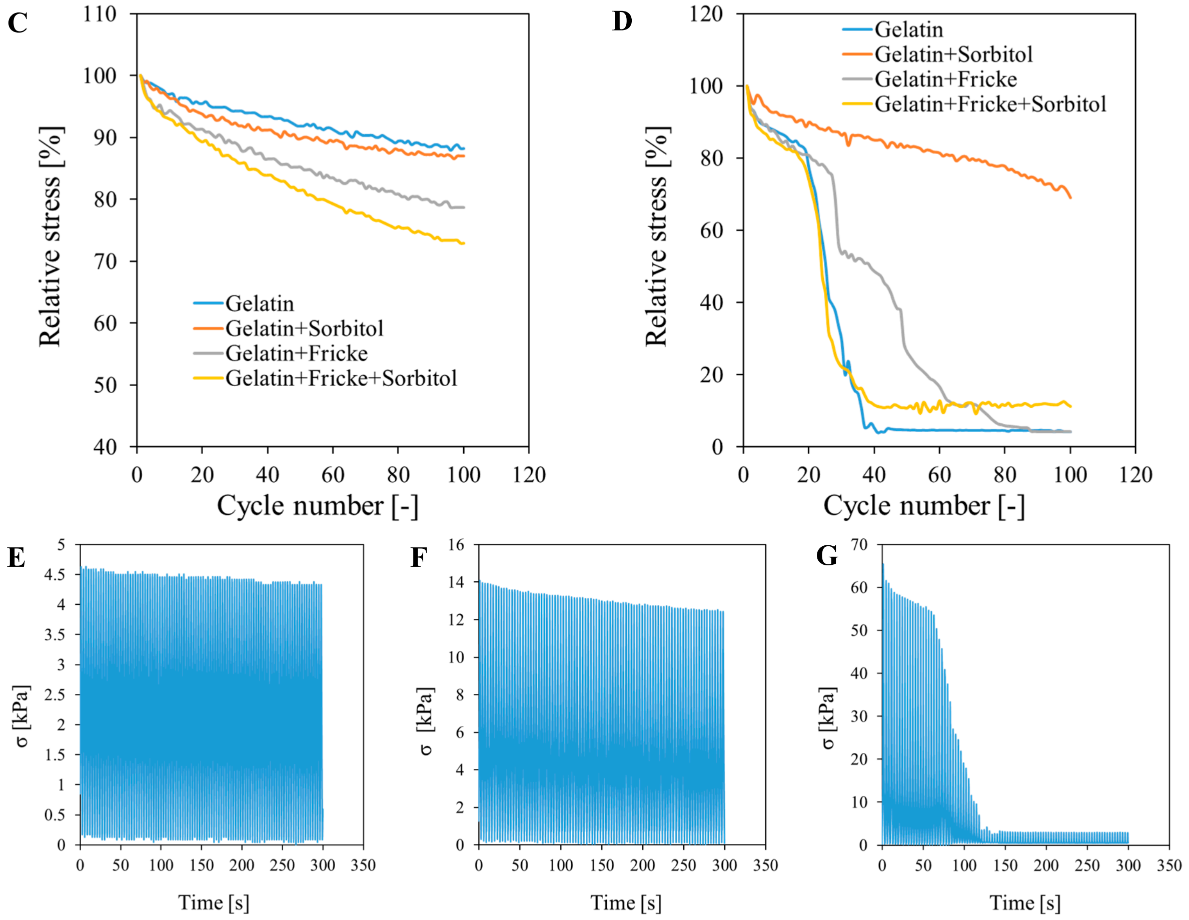

Supplementary Figure S4. **A**: Example compression stress-strain (stress: σ [Pa], strain: ε [-]) characteristics of 8% gelatin, 6% gelatin with 23% sorbitol, 7.96% gelatin with Fricke and 5.99% gelatin with 23% sorbitol and Fricke. The concentrations of the Fricke solution components in the samples were 50 mM H_2SO_4, 0.5 mM FAS, 0.165 mM XO. Measurements were performed 24 h after preparation. The dependence of relative stress as a function of compression cycle number is shown in **B** (20% strain), **C** (40% strain) and **D** (60% strain). Example stress as a function of time for gelatin is shown in **E** (20% strain), **F** (40% strain) and **G** (60% strain).

Supplementary Table S1. The mean values of Young's modulus (E=σ/ε, where σ denotes stress and ε denotes strain), deformation at break and compressive strength of the tested Fricke-XO-Gelatin with sorbitol gels. The samples tested were 8% gelatin, 6% gelatin with 23% sorbitol, 7.96% gelatin with Fricke and 5.99% gelatin with Fricke and 23% sorbitol. The concentrations of the Fricke solution components in the samples were 50 mM H_2SO_4, 0.5 mM FAS, 0.165 mM XO. Measurements were performed 24 h after preparation.

Sample	Compression test		
	Young Modulus [kPa]	Deformation at break [-]	Compressive strength [kPa]
Gelatin	24.9±0.97	0.64±0.014	87.9±4.6
Gelatin+Sorbitol	19.5±0.59	0.67±0.011	108.1±3.8
Gelatin+Fricke	21.1±0.47	0.66±0.003	70.5±4.7
Gelatin+Sorbitol+Fricke	20.5±2.81	0.71±0.017	107.1±15.1

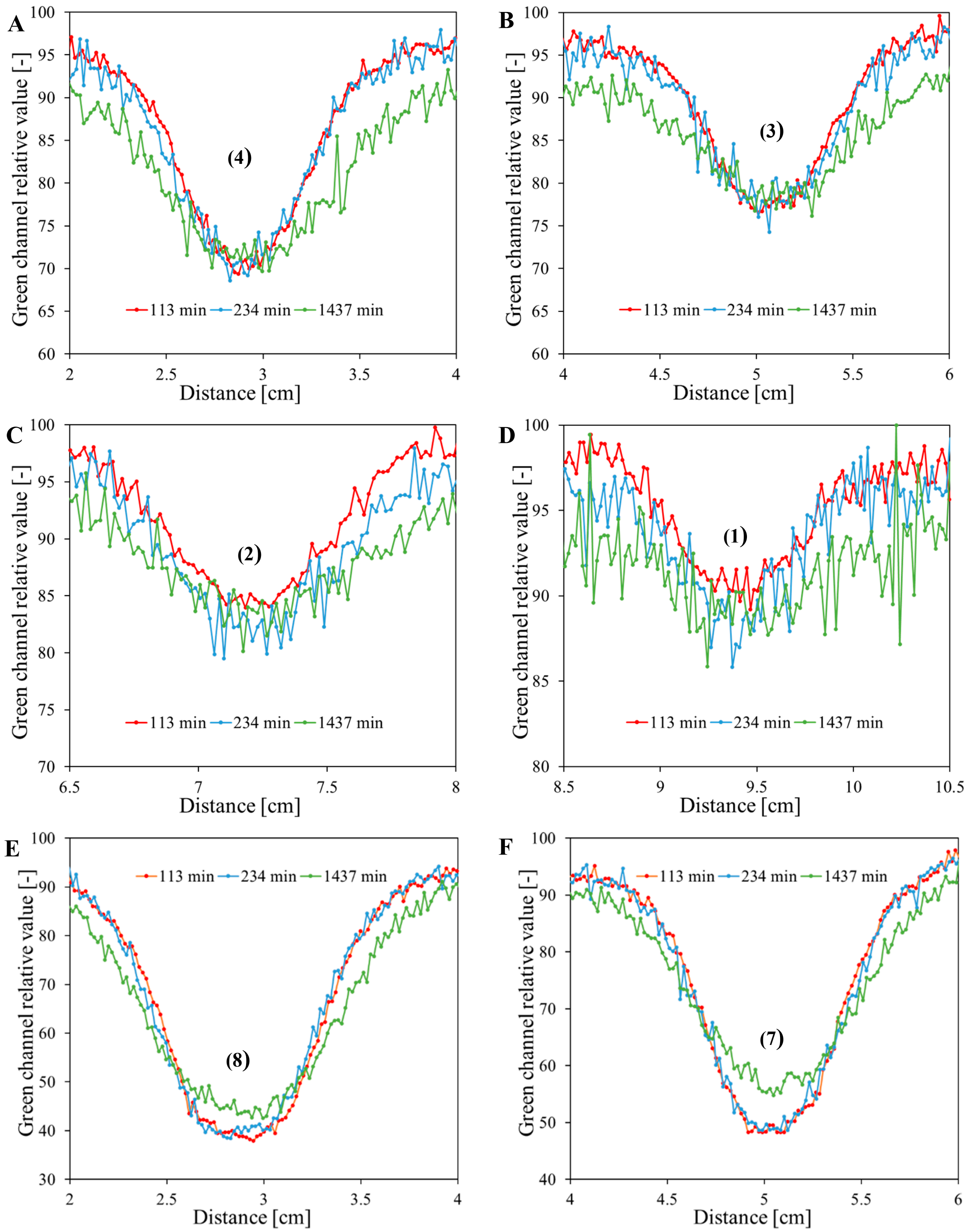
A
(4)
113 min
234 min
1437 min
Green channel relative value [-]
Distance [cm]
B
(3)
113 min
234 min
1437 min
Green channel relative value [-]
Distance [cm]
C
(2)
113 min
234 min
1437 min
Green channel relative value [-]
Distance [cm]
D
(1)
113 min
234 min
1437 min
Green channel relative value [-]
Distance [cm]
E
(8)
113 min
234 min
1437 min
Green channel relative value [-]
Distance [cm]
F
(7)
113 min
234 min
1437 min
Green channel relative value [-]
Distance [cm]

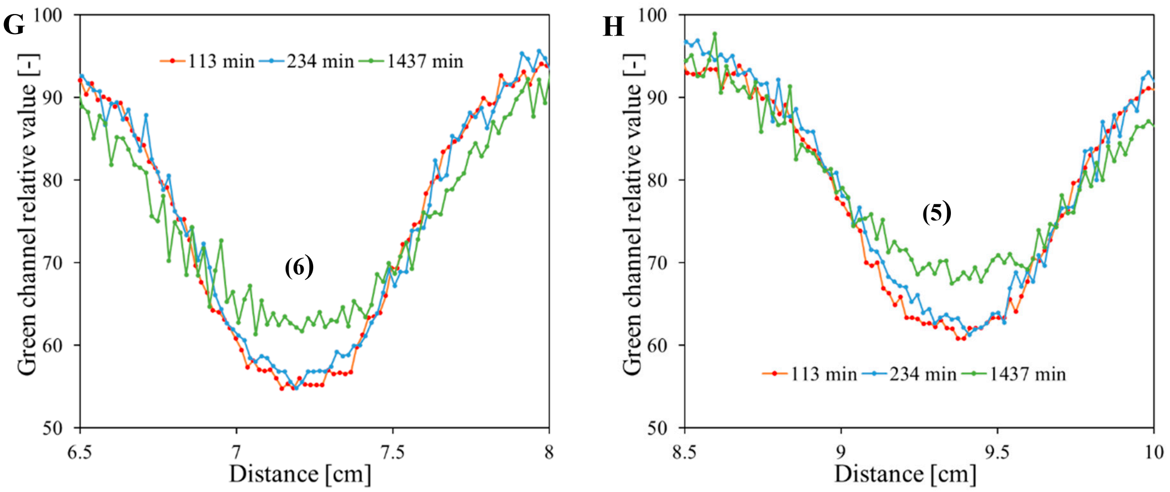

Supplementary Figure S5. Profiles determined across the color stripes obtained after irradiation of the Fricke-XO-Gelatin with sorbitol sample with the following MU values: 250 (**A**), 500 (**B**), 750 (**C**), 1000 (**D**), 1500 (**E**), 2000 (**F**), 2500 (**G**), and 4000 (**H**). Numbers 1-8 correspond to irradiated areas in Figure 6B.

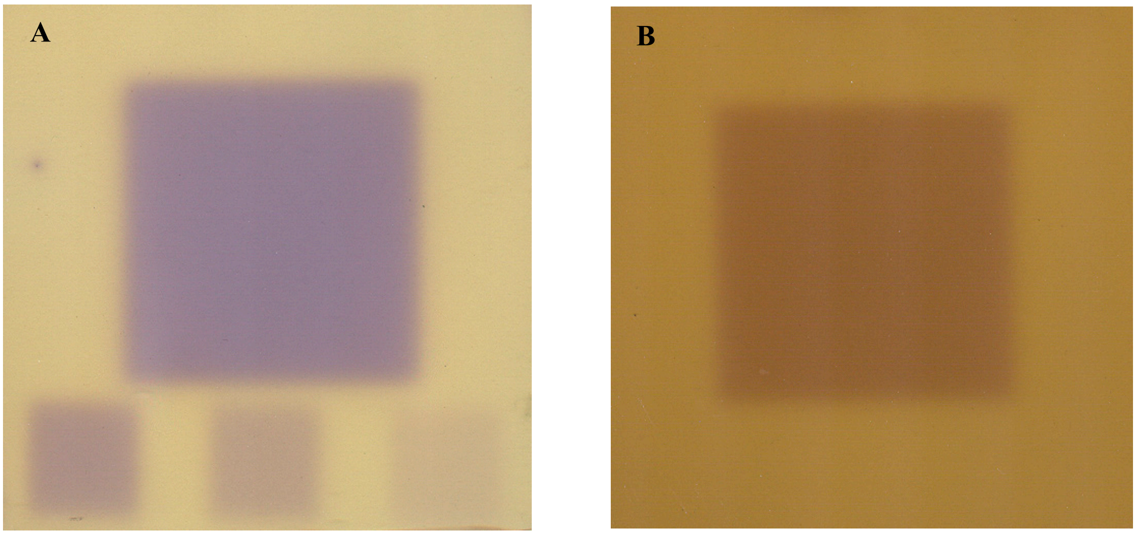

Supplementary Figure S6. Scans of thin Fricke-XO-Gelatin with sorbitol dosimeter (A) and bolus (B) samples irradiated according to the treatment plan. Scans were obtained 80 min (bolus) and 94 min (thin dosimeter) after irradiation.
